# Advancing statistical treatment of photolocomotor behavioral response study data

**DOI:** 10.1371/journal.pone.0300636

**Published:** 2024-05-21

**Authors:** Natalie Mastin, Luke Durell, Bryan W. Brooks, Amanda S. Hering

**Affiliations:** 1 Department of Statistical Science, Baylor University, Waco, TX, United States of America; 2 Department of Environmental Science, Baylor University, Waco, TX, United States of America; 3 Institute of Biomedical Studies, Baylor University, Waco, TX, United States of America; Oregon State University, UNITED STATES

## Abstract

Fish photolocomotor behavioral response (PBR) studies have become increasingly prevalent in pharmacological and toxicological research to assess the environmental impact of various chemicals. There is a need for a standard, reliable statistical method to analyze PBR data. The most common method currently used, univariate analysis of variance (ANOVA), does not account for temporal dependence in observations and leads to incomplete or unreliable conclusions. Repeated measures ANOVA, another commonly used method, has drawbacks in its interpretability for PBR study data. Because each observation is collected continuously over time, we instead consider each observation to be a function and apply functional ANOVA (FANOVA) to PBR data. Using the functional approach not only accounts for temporal dependency but also retains the full structure of the data and allows for straightforward interpretation in any subregion of the domain. Unlike the traditional univariate and repeated measures ANOVA, the FANOVA that we propose is nonparametric, requiring minimal assumptions. We demonstrate the disadvantages of univariate and repeated measures ANOVA using simulated data and show how they are overcome by applying FANOVA. We then apply one-way FANOVA to zebrafish data from a PBR study and discuss how those results can be reproduced for future PBR studies.

## 1 Introduction

In photolocomotor behavioral response (PBR) studies, researchers utilize known brightness preference/avoidance in fish in order to compare the behavior of treatment groups in alternating light/dark conditions over time. This is also often called the “light/dark transition test.” PBR studies have become increasingly prevalent over the past two decades in pharmacological and toxicological research due to their useful indication of the behavioral effect of chemical compounds and potential underlying mode of action. In *Google Scholar*, when the keywords “fish AND locomotor AND behavioral response” are searched, over 90,000 works are returned. When the same keywords are searched in both *PubMed* and *Scopus*, over 15,000 works are represented. When the keywords “fish AND light/dark AND transition” are searched across all three search engines, over 39,000 works are listed. The widespread presence of these studies reflects the fundamental importance of behavioral effects when examining biological activities of chemicals. In fact, the zebrafish model is increasingly used in basic biomedical and environmental research and translational applications because of societal animal welfare concerns and due to governmental mandates that are moving away from use of rodent models. Behavioral changes reflect physiological changes and can impact population-level development, thus acting as an effective tool for assessing the environmental impact of chemical contaminants [1] and behavioral phenotypes during drug discovery [2].

### 1.1 Standard statistical methods used in PBR studies

The standard model used to analyze the behavioral effects of chemical concentrations in the PBR literature is univariate analysis of variance (ANOVA). Responses, such as swim speed, are averaged for each observation either over the entire domain or a section of the domain, and the averages are then used in subsequent analysis. The most common strategy is to calculate the average of the response variable across the entire domain, regardless of light/dark period [3–5]. It is also common for univariate ANOVA to be repeated twice, once for the measurements in the light periods and once for those in the dark periods [6–8]. Alternatively, it may be repeated several times for each individual light/dark period itself [9–11]. When assumptions for univariate one-way ANOVA are not met, Fisher’s Exact test [12] or the nonparametric Kruskal-Wallis test [4, 13, 14] are often used. Other approaches include repeated measures ANOVA [15, 16]; multiple *t*-tests for each level of the factor against the control group [17]; *t*-tests for every time point [18]; and a Kolmogorov-Smirnov test comparing the area under the curve (AUC) of differential entropy between treatment and control groups [14, 19].

**Fig 1:**
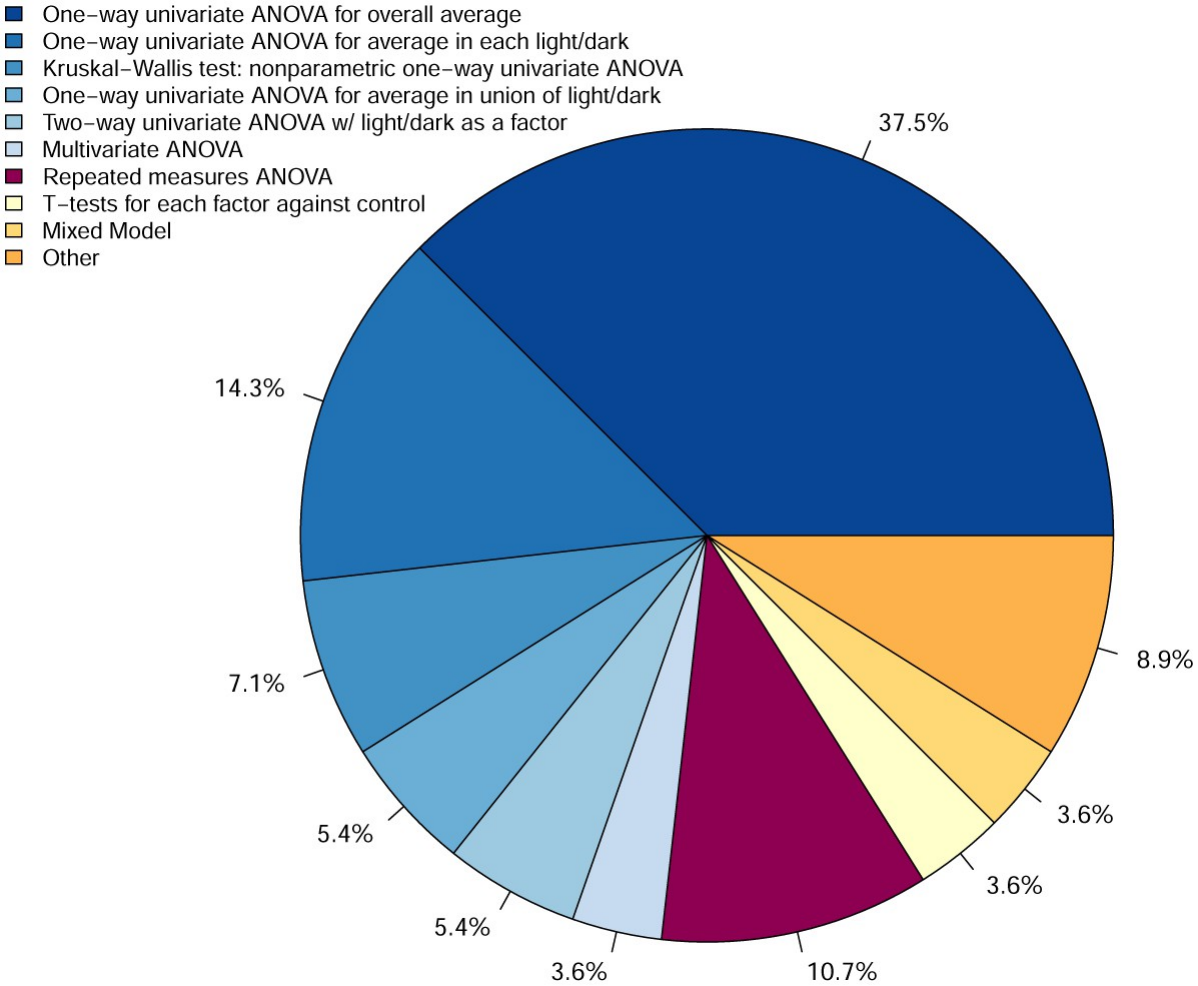
Pie chart representing survey of methods used to analyze PBR study data. Methods represented in shades of blue are variations of traditional ANOVA.

We surveyed over fifty papers in the PBR literature, and while these are not necessarily representative of the entire population of PBR studies, even in this small sample, many different statistical methods to analyze PBR data are represented. These papers were selected based on the expertise of our colleagues who use them for reference in their work. Fig 1 shows ten different categories of methods being used to analyze PBR data in these papers with the majority being a type of one-way ANOVA in which measurements are averaged for each fish. This inconsistency makes comparing study results challenging, if not impossible. Furthermore, [20] mention that there are no available publications comparing the different statistical methods for behavioral studies of zebrafish and recognizes that handling time series data requires models with greater complexity. Over 80% of the papers that we surveyed did not account for the temporal dependency of PBR data, losing useful information about the observations.

Because the data in PBR studies are recorded over time, each individual fish’s observations are dependent. By averaging across the domain and applying univariate ANOVA, the variability within each individual is not accounted for, thereby underestimating the error in the model. This could result in misleading conclusions, as we demonstrate in this paper. Because repeated measures ANOVA models time as a factor, it accounts for individual variability across the domain and is a more favorable way to handle PBR data. However, it is difficult to interpret the interaction between treatment groups and time. When the interaction is significant, post hoc tests must be performed at every time point to determine where in time the differences in treatment levels occur. Most studies that use repeated measures ANOVA test the average differences of the treatment levels in each light/dark period rather than the differences at each time point [21, 22]. Because the raw data are not consistently made publicly available for each PBR study, it is difficult to assess the effects of the statistical methods used on the conclusions drawn. In spite of the fact that many statistical methods are being used in PBR literature, none of the standard methods involve treating the observations as continuous functions, as we propose in this paper. This may occur because once a methodology has been established, it gains momentum, and many researchers continue to use similar methods. Furthermore, functional data analysis (FDA) itself is still a developing branch of statistics, so we hope to encourage researchers to adopt this new paradigm.

### 1.2 The functional approach

**Fig 2:**
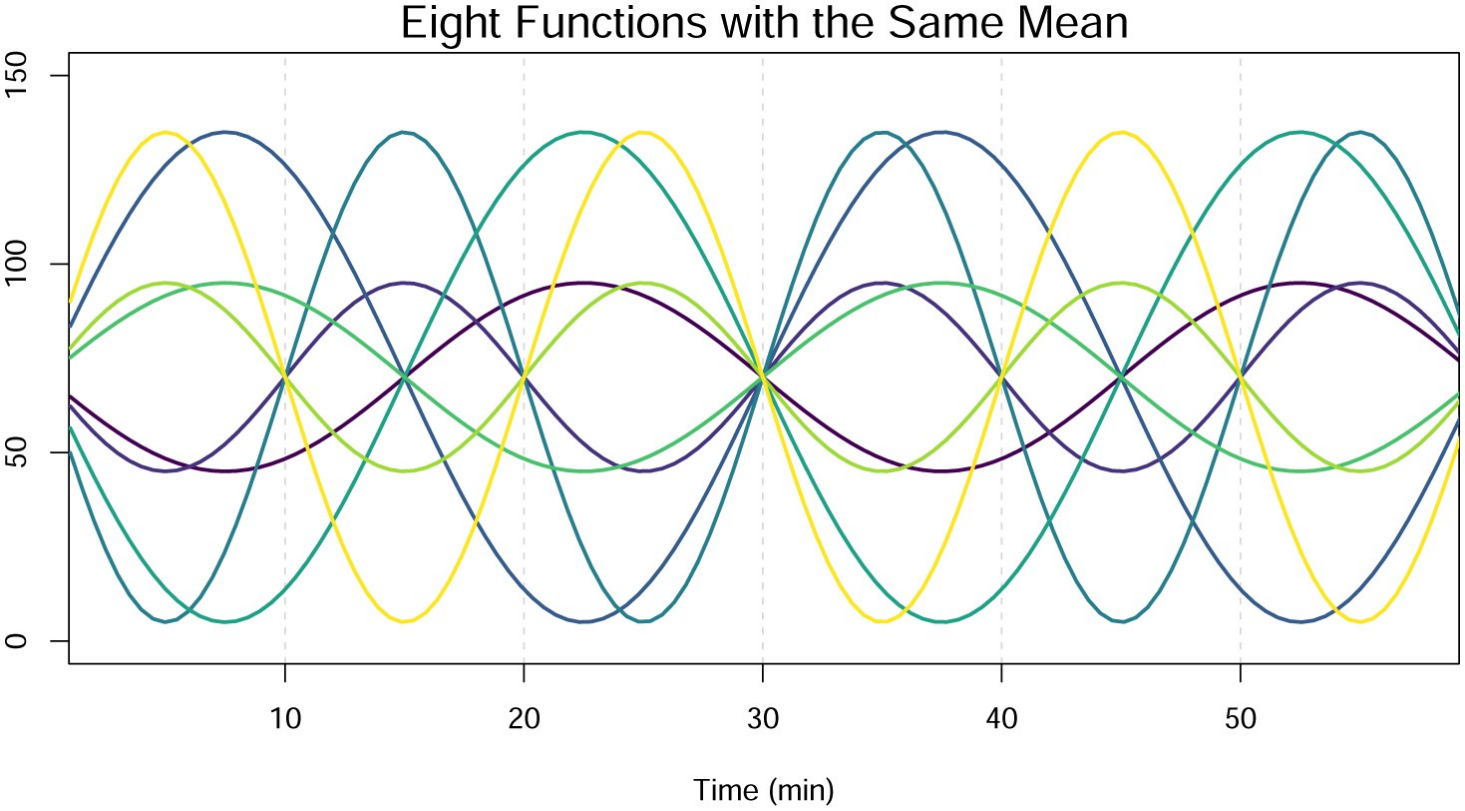
Eight functions that share an overall mean of 70 across their domain.

FDA is a branch of statistical science that deals with data observed over a continuous domain, such as time, and can be respresented as smooth functions [23, 24]. FDA assumes that the data are smooth, naturally accounting for the dependence across the domain. Data in PBR studies are functional in their structure, making FDA the proper approach to use in these studies. Because there are an infinite number of ways that functions can differ and yet still share the same mean, information is lost by averaging across the domain and applying univariate ANOVA. For example, Fig 2 shows eight different functions that share the same mean over their domain, and their shapes clearly differ. If univariate ANOVA were to be performed on the means of these functions, no difference would be found. Similarly, the AUC used in [14, 19] is a summary value and suffers from the same problem in that equal values of AUC can arise from two functions with very different patterns. However, functional ANOVA (FANOVA) would detect a difference in the functions. Results obtained using the functional approach, unlike the univariate approach, are both valid and straightforward to interpret.

In this paper, we present a framework for implementing one-way FANOVA that has minimal assumptions and demonstrate how it can be implemented in PBR study datasets. In Section 2, we review the methodology for univariate ANOVA and repeated measures ANOVA and present functional ANOVA. For each method, we define its models, hypotheses, tests, and assumptions. The FANOVA that we recommend uses a nonparametric permutation test for an *F*-based test statistic and bootstrapped pointwise confidence intervals for the estimated functional effects and contrasts. In Section 3, we illustrate the most extreme possible consequences of applying univariate or repeated measures ANOVA to PBR study data in a simulated example, and we compare these results with one-way FANOVA. Section 4 details a real example using PBR study data and shows how the functional representation of the data provides a seamless analysis of the startle response when brightness conditions change by constructing acceleration functions. Section 5 presents concluding remarks.

## 2 Methodology

In this section, we describe the methodology for traditional univariate ANOVA, repeated measures ANOVA, and FANOVA. To streamline the presentation, we present the balanced one-way case with equal sample sizes in each treatment group.

### 2.1 Univariate ANOVA

In this section, we review the univariate ANOVA model, hypotheses, *F*-statistic, and assumptions. Traditional analysis of variance became widely known after being presented in the classic text by Fisher [25].

#### 2.1.1 Univariate ANOVA model

The response, *y*_*ij*_, is the observed value of subject *j* at level *i* of factor *A*. The one-way univariate ANOVA model is

$$\begin{array}{c} y_{ij}=\mu +\alpha_{i}+\epsilon_{ij}, \end{array}$$ (1)

where *μ* is the overall mean response across the domain; *α*_*i*_ is the treatment effect of factor *A* with levels *i* = 1, …, *a*; and *ϵ*_*ij*_ is the subject effect for *j* = 1, …, *n*_*i*_ replicates. In the balanced case, *n*_*i*_ = *n*.

#### 2.1.2 Univariate ANOVA hypotheses

The following set of hypotheses is used to test the equality of the treatment effect of factor *A*:

$$\begin{array}{cc} H_{0} & :\alpha_{1}=\cdots =\alpha_{a}=0,\\ \text{vs.}\;H_{1} & :\alpha_{i}\neq 0,\;\text{for}\;\text{some}\;i. \end{array}$$ (2)

If *H*_0_ is rejected, then we conclude that there is evidence to support the presence of at least one treatment effect, and post hoc tests are performed on the following hypotheses to determine which pairs of treatment effects differ:

$$\begin{array}{cc} H_{0,\alpha_{i}-\alpha_{i^{\prime }}} & :\alpha_{i}-\alpha_{i^{\prime }}=0,\;\text{for}\;i\neq i^{\prime },\\ \text{vs.}\;H_{1,\alpha_{i}-\alpha_{i^{\prime }}} & :\alpha_{i}-\alpha_{i^{\prime }}\neq 0,\;\text{for}\;i\neq i^{\prime }. \end{array}$$ (3)

#### 2.1.3 Univariate *F*-Test

The parameters in the model are estimated as follows:

$$\begin{array}{cc} \hat{\mu } & ={\bar{y}}_{..},\;\text{and}\\ {\hat{\alpha }}_{i} & ={\bar{y}}_{i.}-{\bar{y}}_{..}, \end{array}$$

where

$$\begin{array}{cc} {\bar{y}}_{..} & =\frac{1}{an}\sum_{i=1}^{a}\sum_{j=1}^{n}y_{ij},\;\text{and}\\ {\bar{y}}_{i.} & =\frac{1}{n}\sum_{j=1}^{n}y_{ij}\;. \end{array}$$

Then, the sums of squares due to the hypothesis (SSA) and error (SSE) are calculated as

$$\begin{array}{c} SSA=n\sum_{i=1}^{a}[{\bar{y}}_{i.}-{\bar{y}}_{..}{]}^{2}=n\sum_{i=1}^{a}{\hat{\alpha }}_{i}^{2},\;\text{and} \end{array}$$ (4)

$$\begin{array}{c} SSE=\sum_{i=1}^{a}\sum_{j=1}^{n}[{\bar{y}}_{ij}-{\bar{y}}_{i.}{]}^{2} \end{array}$$ (5)

The univariate *F*-statistic is

$$\begin{array}{c} F^{obs}=\frac{SSA/q_{A}}{SSE/q_{E}}, \end{array}$$ (6)

and the degrees of freedom (df) associated with *SSA* and *SSE* are *q*_*A*_ = *a* − 1 and *q*_*E*_ = *a*(*n* − 1), respectively.

To obtain the critical value for the rejection region, *F*^*crit*^, the (1 − *τ*)^th^ percentile of the *F*_*qA*,*qE*_ distribution for significance level *τ* is calculated. If *F*^*obs*^ > *F*^*crit*^, then reject *H*_0_, and post hoc analyses are performed. Otherwise, we fail to reject *H*_0_.

#### 2.1.4 Univariate ANOVA assumptions

The following assumptions are required to perform univariate ANOVA:

1. The response variable for each factor level is normally distributed.
2. The distributions of the response variable for each level have the same variance.
3. Each observation is independent of the others.

Averaging the PBR values of an individual across the domain and then applying univariate ANOVA to them ignores the fact that values of each individual are dependent as well as the variability of an individual over the course of the experiment. Therefore, the results may be misleading and should consider biological relevance about the differences in the individuals as a whole. Violation of the independence assumption is considered to have the highest impact of all of these assumptions [26].

### 2.2 Repeated measures ANOVA

In this section, a review of repeated measures ANOVA model, hypotheses, and *F*-statistics is given. This exposition follows largely from [27].

#### 2.2.1 Repeated measures ANOVA model

The response, *y*_*ijk*_, represents the response at time *k* from the *j*th subject in level *i* of factor *A*. The repeated measures ANOVA model is

$$\begin{array}{c} y_{ijk}=\mu +\alpha_{i}+\beta_{k}+{\left(\alpha \beta \right)}_{ik}+\pi_{j(i)}+\epsilon_{ijk}, \end{array}$$ (7)

where *μ* is the overall mean response across the domain, and *α*_*i*_ is the fixed effect of factor *A* with levels *i* = 1, …, *a*. Additionally, *β*_*k*_ is the fixed effect of the time point with levels *k* = 1, …, *t* with $\sum_{k=1}^{t}\beta_{k}=0$, and finally, (*αβ*)_*ik*_ is the fixed effect of the interaction between the *i*th level and the *k*th time point, with constraints

$$\sum_{i=1}^{a}{\left(\alpha \beta \right)}_{ik}=\sum_{k=1}^{t}{\left(\alpha \beta \right)}_{ik}=0.$$

The parameters *π*_*j*(*i*)_ are random effects for the *j*th subject at the *i*th level with *j* = 1, …, *n*. The *π*_*j*(*i*)_ are assumed to be independent and normally distributed with mean zero and variance $\sigma_{\pi }^{2}$. Finally, the *ϵ*_*ijk*_ are independent random error terms with $\epsilon_{ijk}∼N\left(0,\sigma_{\epsilon }^{2}\right)$.

#### 2.2.2 Repeated measures ANOVA hypotheses

The following series of hypotheses is used to test the equality of the treatment effect of factor *A* for all of the time points. The first hypothesis tests the significance of the interaction between the factor and time,

$$\begin{array}{cc} H_{0AT}:\; & {\left(\alpha \beta \right)}_{11}=\cdots ={\left(\alpha \beta \right)}_{at}=0,\\ \;\text{vs.}\;H_{1AT}:\; & {\left(\alpha \beta \right)}_{ik}\neq 0,\;\text{for}\;\text{some}\;ik. \end{array}$$ (8)

If *H*_0*AT*_ is rejected, then there is evidence to support the presence of at least one interaction effect between factor *A* and time, and the cell means, *η*_*ik*_ = *μ* + *α*_*i*_ + *β*_*k*_ + (*αβ*)_*ik*_, are then tested for equality based on the following post hoc hypothesis:

$$\begin{array}{cc} H_{0,\eta_{ik}-\eta_{{\left(ik\right)}^{\prime }}} & :\;\eta_{ik}-\eta_{{\left(ik\right)}^{\prime }}=0,\;\text{for}\;ik\neq {\left(ik\right)}^{\prime },\\ \;\text{vs.}\;H_{1,\eta_{ik}-\eta_{{\left(ik\right)}^{\prime }}} & :\;\eta_{ik}-\eta_{{\left(ik\right)}^{\prime }}\neq 0,\;\text{for}\;ik\neq {\left(ik\right)}^{\prime }. \end{array}$$ (9)

Otherwise, failure to reject *H*_0*AT*_ indicates that there is no significant evidence that the interaction terms differ from zero, leading to tests of the main effects based on the following two hypotheses:

$$\begin{array}{cc} H_{0A} & :\;\alpha_{1}=\cdots =\alpha_{a}=0,\\ \;\text{vs.}\;H_{1A} & :\;\alpha_{i}\neq 0,\;\text{for}\;\text{some}\;i, \end{array}$$ (10)

and

$$\begin{array}{cc} H_{0T} & :\;\beta_{1}=\cdots =\beta_{t}=0,\\ \;\text{vs.}\;H_{1T} & :\;\beta_{k}\neq 0,\;\;\text{for}\;\text{some}\;k. \end{array}$$ (11)

If *H*_0*A*_ or *H*_0*T*_ are rejected, then the following hypotheses are tested:

$$\begin{array}{cc} H_{0,\alpha_{i}-\alpha_{i^{\prime }}} & :\;\alpha_{i}-\alpha_{i^{\prime }}=0,\;\text{for}\;i\neq i^{\prime },\\ \;\text{vs.}\;H_{1,\alpha_{i}-\alpha_{i^{\prime }}} & :\;\alpha_{i}-\alpha_{i^{\prime }}\neq 0,\;\text{for}\;i\neq i^{\prime }, \end{array}$$ (12)

and

$$\begin{array}{cc} H_{0,\beta_{j}-\beta_{k^{\prime }}} & :\;\beta_{k}-\beta_{k^{\prime }}=0,\;\text{for}\;k\neq k^{\prime },\\ \;\text{vs.}\;H_{1,\beta_{j}-\beta_{k^{\prime }}} & :\;\beta_{k}-\beta_{k^{\prime }}\neq 0,\;\text{for}\;k\neq k^{\prime }. \end{array}$$ (13)

When there are a large number of repeated measurements, performing post hoc tests for each individual time point or interaction between factor *A* and time becomes impractical and unwieldy.

#### 2.2.3 Repeated measures *F*-Test

First, the effects are estimated as,

$$\begin{array}{cc} \hat{\mu } & ={\bar{y}}_{\cdot \cdot \cdot },\\ {\hat{\alpha }}_{i} & ={\bar{y}}_{i\cdot \cdot }-{\bar{y}}_{\cdot \cdot \cdot },\\ {\hat{\beta }}_{k} & ={\bar{y}}_{\cdot \cdot k}-{\bar{y}}_{\cdot \cdot \cdot },\\ {\left(\hat{\alpha \beta }\right)}_{ik} & ={\bar{y}}_{i\cdot k}-{\bar{y}}_{i\cdot \cdot }-{\bar{y}}_{\cdot \cdot k}+{\bar{y}}_{\cdot \cdot \cdot },\;\text{and}\;\\ {\hat{\pi }}_{j(i)} & ={\bar{y}}_{ij\cdot }-{\bar{y}}_{i\cdot \cdot }, \end{array}$$

where

$$\begin{array}{cc} {\bar{y}}_{\cdot \cdot \cdot } & =\frac{1}{ant}\sum_{i=1}^{a}\sum_{j=1}^{n}\sum_{k=1}^{t}y_{ijk},\\ {\bar{y}}_{i\cdot \cdot } & =\frac{1}{nt}\sum_{j=1}^{n}\sum_{k=1}^{t}y_{ijk},\\ {\bar{y}}_{\cdot \cdot k} & =\frac{1}{an}\sum_{i=1}^{a}\sum_{j=1}^{n}y_{ijk},\\ {\bar{y}}_{i\cdot k} & =\frac{1}{n}\sum_{j=1}^{n}y_{ijk},\;\text{and}\;\\ {\bar{y}}_{ij\cdot } & =\frac{1}{t}\sum_{k=1}^{t}y_{ijk}. \end{array}$$

Table 1 displays the sum of squares and degrees of freedom for each source of variation.

**Table 1:** Sums of squares and degrees of freedom for repeated measures ANOVA.

Source	DF	Sum Sq
Factor *A*	*a* − 1	*SSA*
Subjects(Factor *A*)	*a*(*n* − 1)	*SSS*(*A*)
Time	*t* − 1	*SST*
Factor *A*×Time	(*a* − 1)(*t* − 1)	*SSAT*
Residual	*a*(*n* − 1)(*t* − 1)	*SSE*

The sums of squares are calculated as

$$\begin{array}{ccc} SSA=t\sum_{i=1}^{a}n{\left[{\bar{y}}_{i\cdot \cdot }-{\bar{y}}_{\cdots }\right]}^{2} & & =t\sum_{i=1}^{a}n{\hat{\alpha }}_{i}^{2}, \end{array}$$ (14)

$$\begin{array}{ccc} SSS(A)=t\sum_{i=1}^{a}\sum_{j=1}^{n}{\left[{\bar{y}}_{ij\cdot }-{\bar{y}}_{i\cdot \cdot }\right]}^{2} & & =t\sum_{i=1}^{a}\sum_{j=1}^{n}{\hat{\pi }}_{j(i)}^{2}, \end{array}$$ (15)

$$\begin{array}{ccc} SST=an\sum_{k=1}^{t}{\left[{\bar{y}}_{\cdot \cdot k}-{\bar{y}}_{\cdots }\right]}^{2} & & =an\sum_{k=1}^{t}{\hat{\beta }}_{k}^{2}, \end{array}$$ (16)

$$\begin{array}{ccc} SSAT=\sum_{i=1}^{a}\sum_{j=1}^{n}\sum_{k=1}^{t}{\left[{\bar{y}}_{i\cdot k}-{\bar{y}}_{i\cdot \cdot }-{\bar{y}}_{\cdot \cdot k}+{\bar{y}}_{\cdots }\right]}^{2} & & =\sum_{i=1}^{a}\sum_{j=1}^{n}\sum_{k=1}^{t}{\left(\hat{\alpha \beta }\right)}_{ik}^{2},\;\text{and} \end{array}$$ (17)

$$\begin{array}{c} SSE=\sum_{i=1}^{a}\sum_{j=1}^{n}\sum_{k=1}^{t}{\left[y_{ijk}-{\bar{y}}_{i\cdot k}-{\bar{y}}_{ij\cdot }+{\bar{y}}_{i\cdot \cdot }\right]}^{2}. \end{array}$$ (18)

Eqs 14, 16 and 17 are the sums of squares needed to test the hypotheses in Eqs 8, 10 and 11, respectively, while Eq 18 is the SSE. The *F*-statistic for testing for differences among groups is given by

$$\begin{array}{c} F_{A}=\frac{MSA}{MSSA}=\frac{SSA/(a-1)}{SSSA/a(n-1)} \end{array}$$ (19)

with *a* − 1 and *a*(*n* − 1) numerator and denominator df, respectively. This test requires the assumption that the within-group covariance matrices are equal. The *F*-statistic for testing differences among time points is given by

$$\begin{array}{c} F_{T}=\frac{MST}{MSE}=\frac{SST/(t-1)}{SSE/[a(n-1)(t-1)]} \end{array}$$ (20)

with *t* − 1 and *a*(*n* − 1)(*t* − 1) numerator and denominator df, respectively. Similarly, the *F*-statistic for testing the significance of the factor *A* × time interaction is given by

$$\begin{array}{c} F_{AT}=\frac{MSAT}{MSE}=\frac{SSAT/[(a-1)(t-1)]}{SSE/[a(n-1)(t-1)]} \end{array}$$ (21)

with (*a* − 1)(*t* − 1) numerator and *a*(*n* − 1)(*t* − 1) denominator df. Both of the prior tests require the assumption that the within-group covariance matrices are equal and that the variances of the differences between variables are equal.

#### 2.2.4 Repeated measures ANOVA assumptions

The following assumptions are required to perform repeated measures ANOVA:

1. The response variable for each factor level is normally distributed.
2. All *ant* observations are independent.
3. The within-group covariance matrices are equal.
4. The variances of the pairwise differences between all combinations of groups are equal; i.e., *Var*(*y*_*ijk*_ − *y*_*i*′*jk*_) is constant for all *i* ≠ *i*′, and *Var*(*y*_*ijk*_ − *y*_*ijk*′_) is constant for all *k* ≠ *k*′. This is usually referred to as the sphericity assumption.

Because time is treated as a factor in repeated measures ANOVA, it is a valid way to analyze PBR study data, but there are several disadvantages to using this approach. It is difficult to interpret the interaction between groups and time. The assumptions of normality, within-group homogeneity of variance, and sphericity are often violated and can bias results. For example, [28] showed that if the sphericity assumption is violated, then the true type I error rate is positively biased, causing excessive false rejection of the null hypotheses for the hypotheses in Eqs 8 and 11. Additionally, [29] notes that even repeated measures ANOVA does not account for temporal autocorrelation between measurements, which is likely present in PBR data, leading to lower power. This limitation is greatest when adjacent time points have very similar responses, which is the case for most of the adjacent time points in PBR study data. Finally, due to the number of repeated measurements over time in PBR data, post hoc analyses require many tests. Functional ANOVA, however, does not have these disadvantages. The functional ANOVA that we present makes minimal assumptions and provides simple interpretations and post hoc analyses.

### 2.3 FANOVA

In this section, we present the one-way FANOVA model, hypotheses, and *F*-based statistics. One-way FANOVA is described in [24, 30]. We also present a nonparametric approach to testing these hypotheses that relies on very few assumptions.

#### 2.3.1 FANOVA model

The functional response, *y*_*ij*_(*t*), is defined over a domain T, with *i* corresponding to the levels of factor *A* and *j* corresponding to the subject. In this case, t∈T, where T represents an interval of time, and *t* represents a specific point in time. The one-way FANOVA model is

$$\begin{array}{c} y_{ij}\left(t\right)=\mu \left(t\right)+\alpha_{i}\left(t\right)+\epsilon_{ij}\left(t\right),\;t\in T, \end{array}$$ (22)

where *μ*(*t*) is the overall functional mean; *α*_*i*_(*t*) is the functional effect of factor *A* with levels *i* = 1, …, *a*; and *ϵ*_*ij*_(*t*) is the functional subject effect for *j* = 1, …, *n* replicates.

#### 2.3.2 FANOVA hypotheses

The following hypotheses test the equality of the functional effects.

$$\begin{array}{cc} H_{0} & :\alpha_{1}\left(t\right)=\cdots =\alpha_{a}\left(t\right)=0,\;\text{for}\;\text{all}\;t\in T,\\ \text{vs.}\;H_{1} & :\alpha_{i}\left(t\right)\neq 0,\;\text{for}\;\text{some}\;t\in T\;\text{and}\;\text{level}\;i. \end{array}$$ (23)

If *H*_0_ is rejected, then we can conclude that there is evidence of at least one functional effect, and post hoc tests are performed for the following hypotheses to determine which pairs of functional effects differ:

$$\begin{array}{cc} H_{0,\alpha_{i}-\alpha_{i^{\prime }}} & :\alpha_{i}\left(t\right)-\alpha_{i^{\prime }}\left(t\right)=0,\;\text{for}\;\text{all}\;t\in T\;\text{with}\;i\neq i^{\prime },\\ \text{vs.}\;H_{1,\alpha_{i}-\alpha_{i^{\prime }}} & :\alpha_{i}\left(t\right)-\alpha_{i^{\prime }}\left(t\right)\neq 0,\;\text{for}\;\text{some}\;t\in T\;\text{with}\;i\neq i^{\prime }. \end{array}$$ (24)

#### 2.3.3 Smoothing the functions

Before applying FANOVA to the data, the first step is to create functions of the discretized values by taking a weighted sum of basis functions. The fda package in R [31] has many helpful functions for smoothing, and we present a summary of this process [24]. Basis functions are sets of mathematically independent functions, and by taking linear combinations of them, they can be used to approximate any function. The degree to which the data are smoothed rather than interpolated is determined by the number of basis functions. As the number of basis functions increases, the function can be more closely approximated, but this introduces a possibility of overfitting to noise. Interpolation is achieved when the number of basis functions is equal to the number of discrete values recorded in the domain, but this is only desirable when the measurements are errorless. Once the functions have been fit, they can be evaluated across a fine grid of values over the domain.

The first choice to be made in smoothing is which basis set to use. Many basis systems are described in [24]. If possible, the set of basis functions chosen should have features that match those of the functions being approximated. For example, if the data have periodic features, a Fourier basis can achieve a good approximation using fewer basis functions. Fourier bases are most useful when the data have no strong local features, and there is no need to reflect discontinuities in their derivatives. For data with no clear structure, cubic b-splines are the most common choice of approximation system, and they can be engineered to have discontinuity in the functions.

Less important than the choice of basis set is how many basis functions should be used in the approximation. However, the modern way to smooth functions is to use the maximum number of basis functions, or to “saturate” the model, allowing the number of basis functions to match the number of measured values for each observation. This would produce functions that are interpolated and thereby very rough, so a penalty is imposed that controls the trade-off between fit to the data and smoothness. The smoothness of the function is controlled by a smoothing parameter that penalizes its roughness using the square of the second derivative of the estimated functions. The smoothing parameter is chosen by minimizing generalized cross validation of the squared error between the smooth and discretized values. By minimizing the squared error, unusually high values are allowed to be influential, but their impact on the overall analysis is dampened.

The frequency of measurements necessary to construct a continuous function may vary based on the variability of the data. The data should be collected frequently enough to capture important changes in the process at the rate at which they occur. For example, if rapid changes are expected to occur at a temporal scale of less than a minute, data should be collected more frequently than a minute. In order to create a smooth function that represents the true data, features of interest must be captured through the discretized measurements.

#### 2.3.4 Functional *F*-Statistic

Given the smoothed functions, we then estimate the functional effects as

$$\begin{array}{cc} \hat{\mu }\left(t\right) & ={\bar{y}}_{..}\left(t\right),\;\text{and}\\ {\hat{\alpha }}_{i}\left(t\right) & ={\bar{y}}_{i.}\left(t\right)-{\bar{y}}_{..}\left(t\right), \end{array}$$

where

$$\begin{array}{cc} {\bar{y}}_{..}\left(t\right) & =\frac{1}{an}\sum_{i=1}^{a}\sum_{j=1}^{n}y_{ij}\left(t\right),\;\text{and}\\ {\bar{y}}_{i.}\left(t\right) & =\frac{1}{n}\sum_{j=1}^{n}y_{ij}\left(t\right). \end{array}$$

Then, the functional sums of squares due to the hypothesis (*SSA*(*t*)) and error (*SSE*(*t*)) are calculated as

$$\begin{array}{c} SSA\left(t\right)=n\sum_{i=1}^{a}[{\bar{y}}_{i.}\left(t\right)-{\bar{y}}_{..}\left(t\right){]}^{2}=n\sum_{i=1}^{a}{\hat{\alpha }}_{i}^{2}\left(t\right),\;\text{and} \end{array}$$ (25)

$$\begin{array}{c} SSE\left(t\right)=\sum_{i=1}^{a}\sum_{j=1}^{n}[{\bar{y}}_{ij}\left(t\right)-{\bar{y}}_{i.}\left(t\right){]}^{2}. \end{array}$$ (26)

The general functional *F*-statistic is

$$\begin{array}{c} F^{obs}\left(t\right)=\frac{SSA\left(t\right)/q_{A}}{SSE\left(t\right)/q_{E}},\;t\in T, \end{array}$$ (27)

where the degrees of freedom associated with *SSA*(*t*) and *SSE*(*t*) are *q*_*A*_ = *a* − 1 and *q*_*E*_ = *a*(*n* − 1), respectively. These quantities are the functional versions of the scalar counterparts in one-way univariate ANOVA.

#### 2.3.5 *F*_*FT*_ statistic and permutation test

The functional *F*_*FT*_ test statistic [32] summarizes the variation across levels over the domain through integration, providing a scalar *F*-value and corresponding *p*-value. This overall statistic is

$$\begin{array}{c} F_{FT}^{obs}=\frac{\int_{T}SSA\left(t\right)dt/q_{A}}{\int_{T}SSE\left(t\right)dt/q_{E}}. \end{array}$$ (28)

To approximate the distribution of *F*_*FT*_ under the null hypothesis in Eq 23, the functions are permuted (reassigned to a random level of Factor A) a large number of times, and *F*_*FT*_ is recalculated for each permutation of the functions. The values of *F*_*FT*_ based on the permuted samples are denoted $F_{FT}^{r}$ for *r* = 1, …, *R*. Then, for a given significance level, *τ*, the 100 × (1 − *τ*)^*th*^ percentile of the $F_{FT}^{r}$ values is calculated. If the observed statistic, $F_{FT}^{obs}$, exceeds the critical value, $F_{FT}^{crit}$, then we reject *H*_0_. Otherwise, we fail to reject *H*_0_. A scalar *p*-value can be estimated by calculating the proportion of $F_{FT}^{r}$ permutations that exceed $F_{FT}^{obs}$.

Additional *F*-based statistics have been proposed to summarize the general *F*(*t*) statistic, such as the *L*^2^-norm [33], globalizing-*F* [34], and *F*-max statistics [35]. Any of these *F*-based statistics could be used for this method, but as shown in [36], the *F*_*FT*_ test is very close to the nominal size and is slightly below *τ* = 0.05, resulting in fewer rejections of a true null hypothesis. Furthermore, it has high power under a variety of alternative hypotheses. Because of the fundamental differences in the hypotheses between univariate ANOVA, repeated measures ANOVA, and FANOVA, the power of the three methods cannot be compared to one another; however, the power of the *F*_*FT*_ test compared to the other *F*-based FANOVA tests is explored further in [36]. The only assumption required for the FANOVA method described here is that the individuals are independent of each other.

There are some challenges with implementing FANOVA. Smoothing the curves reduces variability in the data, which underestimates the true error in the FANOVA model, but this underestimation can be reduced by fitting the smooth curves to minimize the error between them and the discretized values, as described in Section 2.3.3. Furthermore, smoothing reduces the impact of outliers in the data, but even with data-driven choices in place, such as using cross-validation to choose the smoothing parameter or choice of basis sets, some subjectivity in smoothing is unavoidable. Thus, a researcher should investigate the effects of smoothing on their conclusions. Secondly, knowledge of a programming language is currently required to implement the nonparametric FANOVA that we present. However, the code that we developed for this analysis is publicly available on the Harvard Dataverse repository [37], and many modifications can be made with a low level of effort to adapt the code to different PBR studies.

## 3 Illustration

To illustrate how traditional ANOVA can mask important features in PBR data, we develop a one-way functional ANOVA example using artificial data for three levels of a factor over a period of sixty minutes. The factor could correspond to a chemical toxicant with three concentration levels, and the response could be the swim speed.

### 3.1 Simulating the data

In this illustration, we simulate 20 observations under each level by adding random variation to the amplitude, period, and phase of sine curves.. For *j* = 1, …, 20 and *t* ∈ [1, 60], let *f*_*i*,*j*_(*t*) represent the functional observation of the *i*^th^ fish observed under level *i* = 1, 2, 3 evaluated at minute *t*. All of the observations can be represented as

$$\begin{array}{c} f_{i,j}\left(t\right)=70+A_{i,j}\;\text{sin}(\frac{\pi t}{R_{i,j}}+\pi P_{i,j})+W_{i}, \end{array}$$ (29)

where *A*_*i*,*j*_ represents variation in the amplitude; *R*_*i*,*j*_ represents variation in the period; *P*_*i*,*j*_ represents variation in the phase; and *W*_*i*_ ∼ iid N(0, *σ*^2^ = 36) represents random individual variation. The distributions for the amplitude, period and phase of each level are shown in Table 2.

**Table 2:** Distributions for random variation in amplitude, period, and phase for each level of the factor.

Function	Amplitude	Period	Phase
*f*_1,*j*_(*t*)	*A*_1,*j*_ ∼ iid Unif(37, 43)	*R*_1,*j*_ ∼ iid N(15, 0.025)	*P*_1,*j*_ ∼ iid N(0, 0.0225)
*f*_2,*j*_(*t*)	*A*_2,*j*_ ∼ iid Unif(17, 23)	*R*_2,*j*_ ∼ iid N(15, 0.025)	*P*_2,*j*_ ∼ iid N(1, 0.0225)
*f*_3,*j*_(*t*)	*A*_3,*j*_ ∼ iid Unif(57, 63)	*R*_3,*j*_ ∼ iid N(10, 0.025)	*P*_3,*j*_ ∼ iid N(1, 0.0225)

For each level, the true average swim pattern can be represented as

$$\begin{array}{c} \bar{f_{i}}\left(t\right)=70+{\bar{A}}_{i}\;\text{sin}(\frac{\pi t}{{\bar{R}}_{i}}+\pi {\bar{P}}_{i}), \end{array}$$ (30)

where ${\bar{A}}_{i}$, ${\bar{R}}_{i}$, and ${\bar{P}}_{i}$ are the averages of the distributions in Table 2 and are given in Table 3. Note that the averages of the mean functions taken across the domain are equal, or

$$\begin{array}{c} \int_{T}\bar{f_{1}}\left(t\right)dt=\int_{T}\bar{f_{2}}\left(t\right)dt=\int_{T}\bar{f_{3}}\left(t\right)dt=70. \end{array}$$

**Table 3:** Average amplitude, period, and phase for the mean swim pattern functions.

Mean Function	Amplitude	Period	Phase
$\bar{f_{1}}\left(t\right)$	${\bar{A}}_{1}=40$	${\bar{R}}_{1}=15$	${\bar{P}}_{1}=0$
$\bar{f_{2}}\left(t\right)$	${\bar{A}}_{2}=20$	${\bar{R}}_{2}=15$	${\bar{P}}_{2}=1$
$\bar{f_{3}}\left(t\right)$	${\bar{A}}_{3}=60$	${\bar{R}}_{3}=10$	${\bar{P}}_{3}=1$

**Fig 3:**
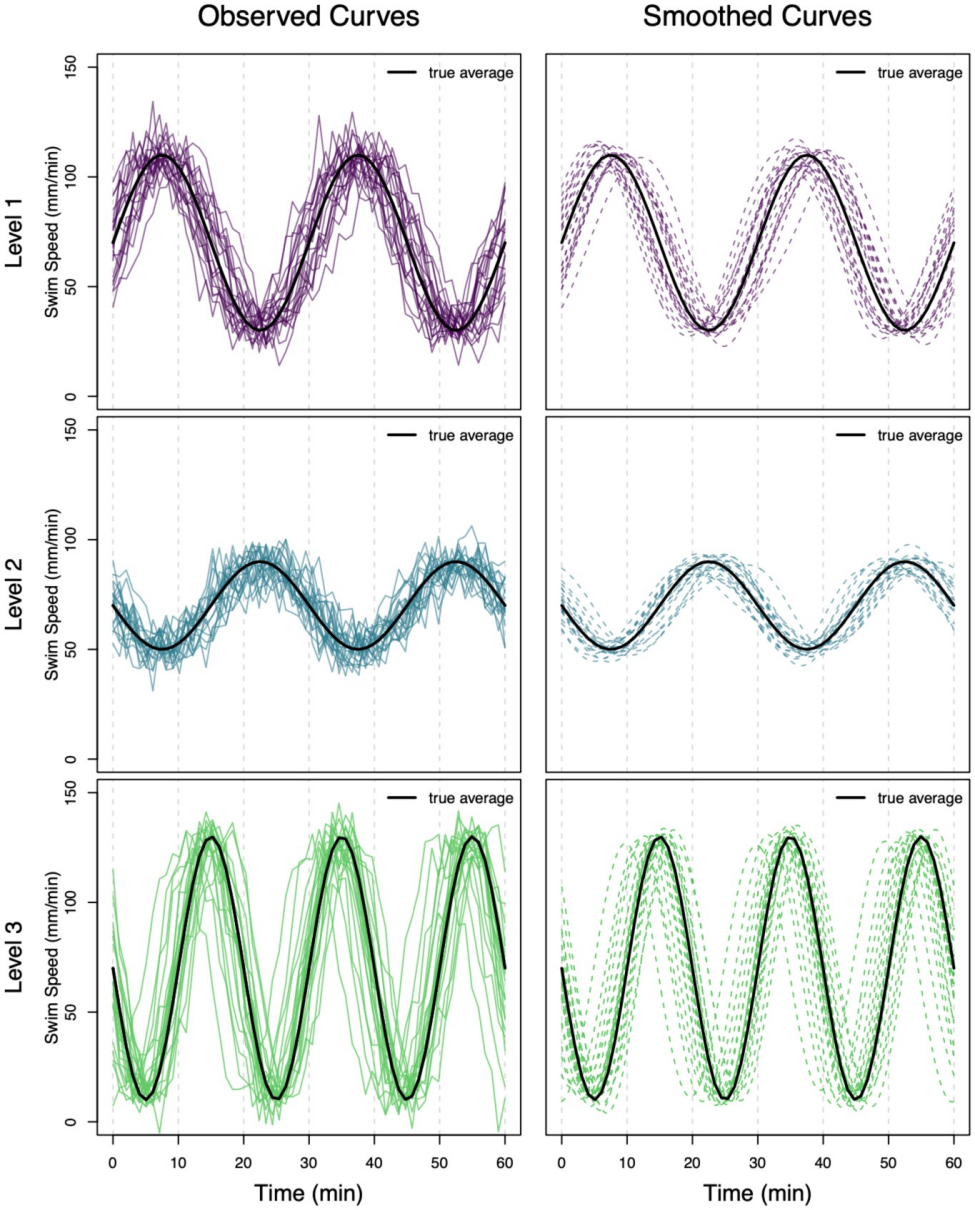
The left side shows swim speed over time of twenty fish simulated under level 1 of the factor (top), level 2 of the factor (middle), and level 3 of the factor (bottom), with the true average function overlaid in black. These are treated as the observed data. The right side shows the corresponding smoothed functions of swim speed.

**Fig 4:**
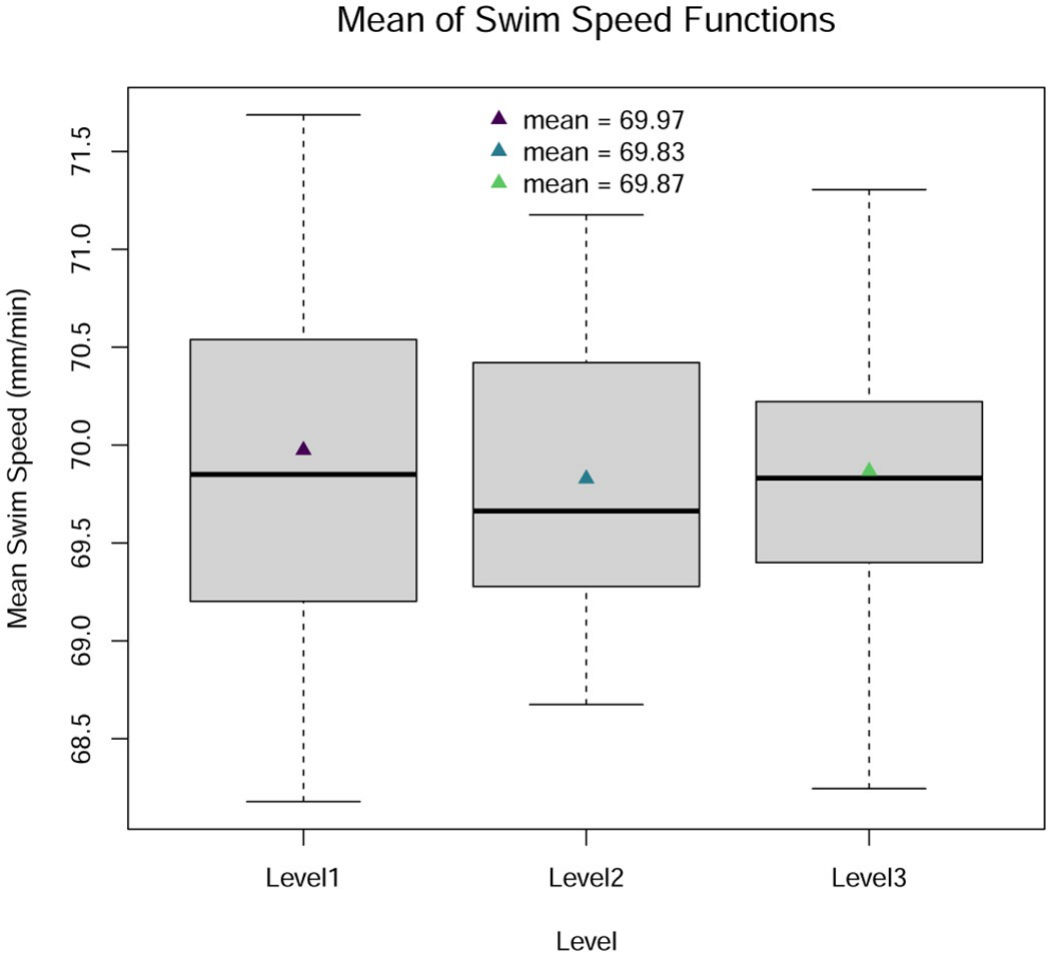
Boxplots of the averages of the functions simulated under three levels of a factor.

The left column of Fig 3 shows twenty functions simulated from Eq 29 for each level. All sets of functions are smoothed prior to analysis with a Fourier basis set with ten basis functions. The Fourier basis system is chosen because the simulated data are periodic, and the number of basis functions is selected to balance interpolation and smoothness of the data. The results of the Fourier smoothing are shown in the right column of Fig 3. For each function, the mean across the domain is calculated, and Fig 4 shows a boxplot of these values. The boxplots have very similar means for each of the three levels even though the function shapes at each level are clearly different.

### 3.2 Univariate and repeated measures ANOVA

If we are interested in determining if there is a difference in the swim speeds of the fish observed under the three levels, then it would problematic to employ univariate ANOVA because reducing each function to its average does not account for the variability in swim speed over time. In this particular case, the true mean for each of the functions in Fig 3 is 70, so the null hypothesis in the univariate ANOVA is true. Therefore, the correct decision is to fail to reject the null hypothesis. Indeed, when we apply a univariate ANOVA to the means of the functions in Fig 4, a large *p*-value is produced, as shown in Table 4, indicating that there is no significant difference in the mean swim speeds of the fish under the three conditions. However, when viewed over the domain, the fish observed under the three levels have very different swim patterns.

**Table 4:** Univariate ANOVA results.

	Df	Sum Sq	Mean Sq	F-value	Pr(>F)
Factor	2	0.23	0.11	0.17	0.8475
Residual	57	38.85	0.68		

Next, we perform a repeated measures ANOVA, and the results are shown in Table 5. We find that the interaction between the factor and time is significant, which is an improvement upon the univariate ANOVA that did not detect any difference between the three levels. Due to the violation the normality and sphericity assumptions, however, these results are inconclusive. Usually when assumptions of a test are not met, a nonparametric method is used, but no such test can be performed in this context. Ignoring the violated assumptions, the significant interaction between the factor and time implies that a post hoc analysis should be performed. In this case, there would be 3 × 60 = 180 post hoc comparisons, and this is a prohibitive number of comparisons to make, so we do not present the post hoc analyses for the repeated measures ANOVA.

**Table 5:** Repeated measures ANOVA results.

	Df	Sum Sq	Mean Sq	F-value	Pr(>F)
Factor	2	13.57	6.79	0.17	0.8475
Fish(Factor)	57	2331.24	40.90		
Time	59	574781.29	9742.06	38.79	<0.0001
Factor×Time	118	1980732.70	16785.87	66.84	<0.0001
Residual	3363	844573.03	251.14		

### 3.3 FANOVA application

**Fig 5:**
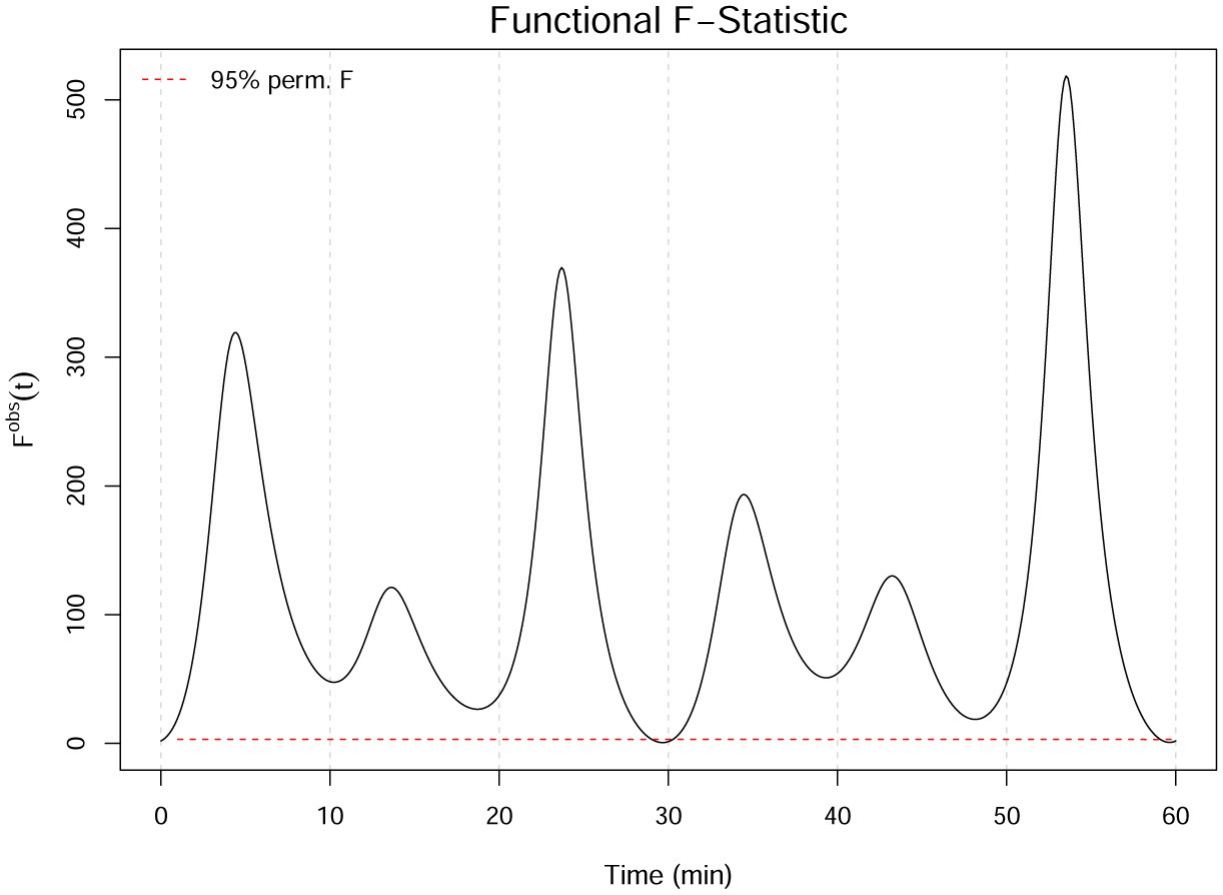
One-way FANOVA *F*^*obs*^(*t*) statistic based on the smoothed dataset. The red dashed line indicates the 95% pointwise percentile calculated from the permutations.

Finally, we apply functional ANOVA to this simulated data. Using 10,000 permutations and a significance level of *τ* = 0.05, the permuted critical value is $F_{FT}^{crit}=2.945$, which is much smaller than the observed value of $F_{FT}^{obs}=77.468$. The *p*-value for $F_{FT}^{obs}$ is zero, meaning that 100% of the permuted test statistic values were less than $F_{FT}^{obs}$. Fig 5 shows the *F*^*obs*^(*t*) statistic from the one-way functional ANOVA results on the smoothed data. The *F*^*obs*^(*t*) statistic is particularly high in regions where the functional means differ the most. FANOVA not only captures the significant difference in the mean swim pattern functions, but it also identifies the approximate locations in the domain where the differences occur.

**Fig 6:**
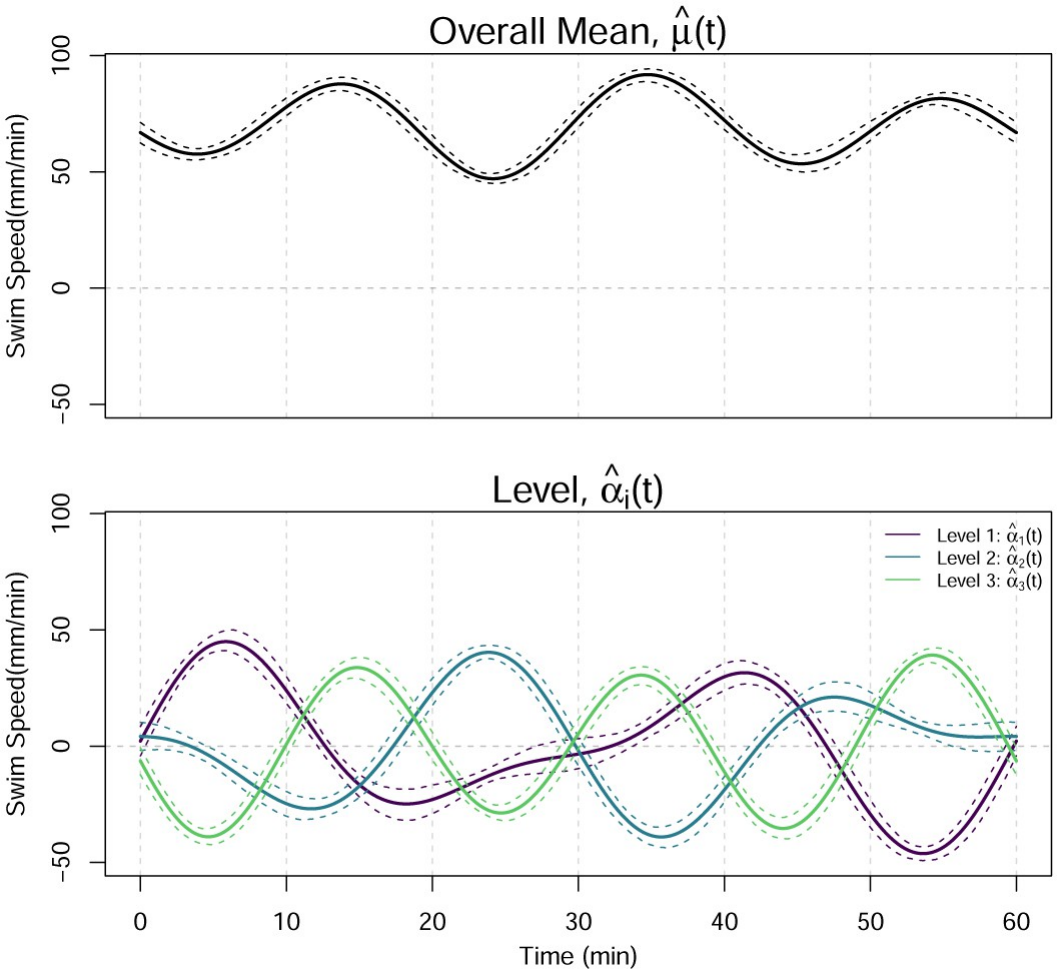
Estimated overall mean effect (top) and factor effect (bottom). Dashed lines are 95% pointwise confidence intervals formed using 1000 bootstrap replicates.

If the overall function mean and differences from this overall mean for each level are of interest, then the estimated functional effects can provide insight, as shown in Fig 6. The top panel of Fig 6 shows the overall functional mean effect, $\hat{\mu }\left(t\right)$, and the bottom panel shows the functional effect of each level of factor *A*, ${\hat{\alpha }}_{i}\left(t\right)$. To form 95% pointwise bootstrapped confidence intervals for each of the estimated functional effects, we sample with replacement 1000 times from the sixty estimated functional subject effects,

$$\begin{array}{c} {\hat{\nu }}_{ij}\left(t\right)=y_{ij}\left(t\right)-\hat{\mu }\left(t\right)-{\hat{\alpha }}_{i}\left(t\right),\;i=1,\ldots 3,\;j=1,\ldots ,20, \end{array}$$

resulting in ${\hat{\nu }}_{ij}^{r}\left(t\right)$ for *r* = 1, …, 1000. These are used to construct bootstrapped observations as

$$\begin{array}{c} y_{ij}^{r}\left(t\right)={\hat{\nu }}_{ij}^{r}\left(t\right)+\hat{\mu }\left(t\right)+{\hat{\alpha }}_{i}\left(t\right),\;i=1,\ldots 3,\;j=1,\ldots ,20. \end{array}$$ (31)

The functional effects, ${\hat{\mu }}^{r}\left(t\right)$ and ${\hat{\alpha }}_{i}^{r}\left(t\right)$, are reestimated based on the bootstrap samples in Eq 31. The difference between the bootstrapped and observed functional effects are calculated, and the 2.5% and 97.5% pointwise percentiles of the 1000 difference functions at each *t* are taken to be the upper and lower bounds of the confidence interval. These confidence intervals can only be interpreted at a point, but they provide a helpful visual reference for the variability of the estimated functional effects. The computational time to construct the permutation test and bootstrap intervals is minimal, requiring less than a minute on a personal laptop computer.

#### 3.3.1 Post hoc analysis

**Fig 7:**
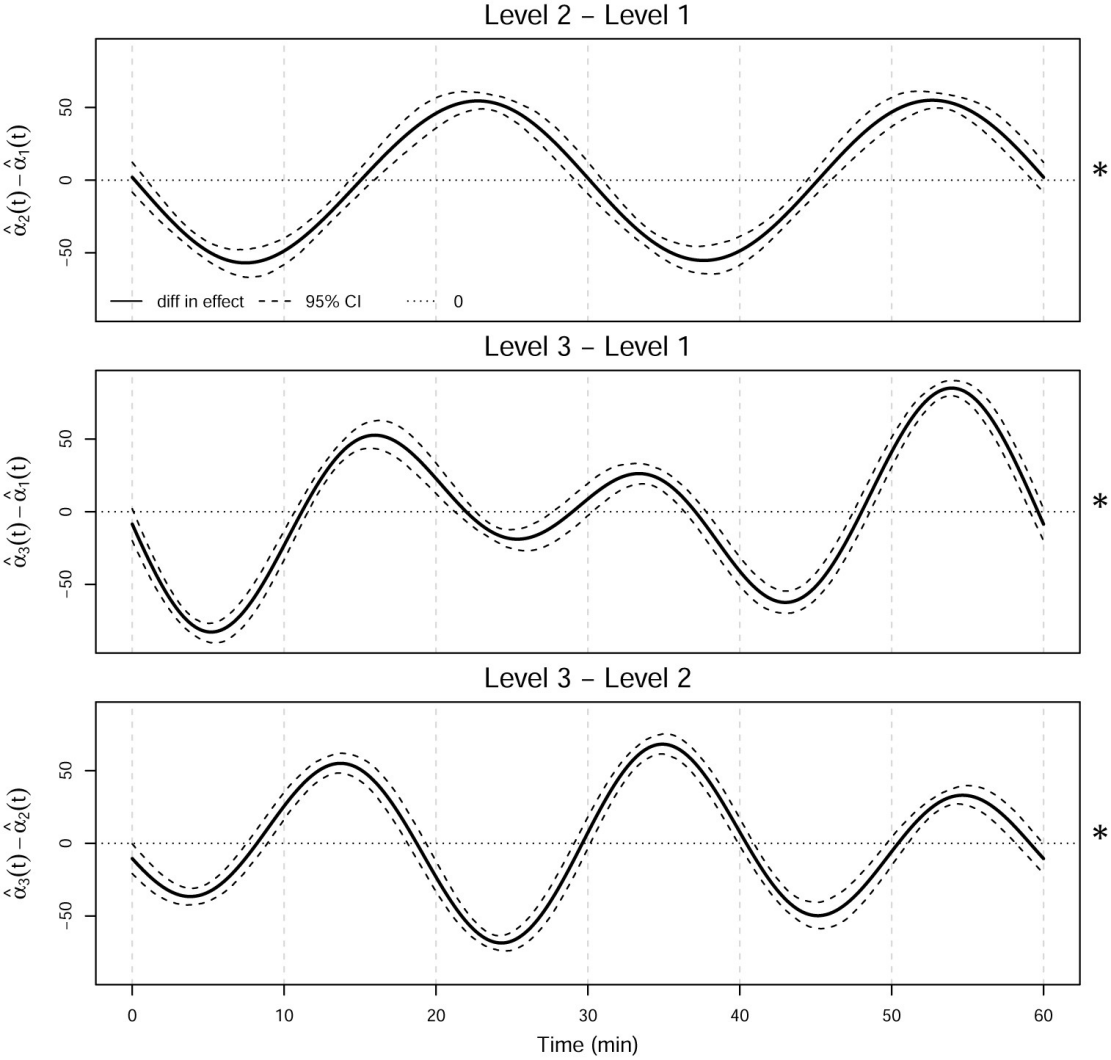
Functional post hoc contrasts for the levels. The dashed line is the pointwise 98.33% confidence interval using 1000 bootstrap replicates. The asterisk on the right indicates significance of the *F*_*FT*_ test for the factor effect.

Once the null hypothesis is rejected, we perform post hoc analyses to determine which pairs of levels differ significantly from one another. The tests employ a Bonferroni correction so that the significance level for each test is the nominal significance level (*τ* = 0.05) divided by the number of tests. With three levels, there are three pairwise comparisons formed by calculating ${\hat{\alpha }}_{i}\left(t\right)-{\hat{\alpha }}_{i^{\prime }}\left(t\right)$ with *i* ≠ *i*′, so the adjusted level of significance is 0.05/3 = 0.017. The 95% pointwise bootstrapped confidence intervals are constructed as described in Section 3.3 with 1000 replicates and are shown in Fig 7. There is a significant difference between the mean swim speeds of each pair of levels.

## 4 Real data example

In this example, we demonstrate one-way FANOVA on a dataset from a PBR study that compares the effects of an environmental toxicant, 1-heptanol, of different concentrations on the swim patterns of zebrafish over alternating lightness conditions [8]. Experimental results examined here were obtained from studies that were approved by the Institutional Animal Care and Use Committee at Baylor University. Swim speed is recorded for a total of fifty minutes, divided into a ten-minute dark acclimation period followed by four alternating light and dark periods, each lasting ten minutes. A measurement is recorded every minute.

**Fig 8:**
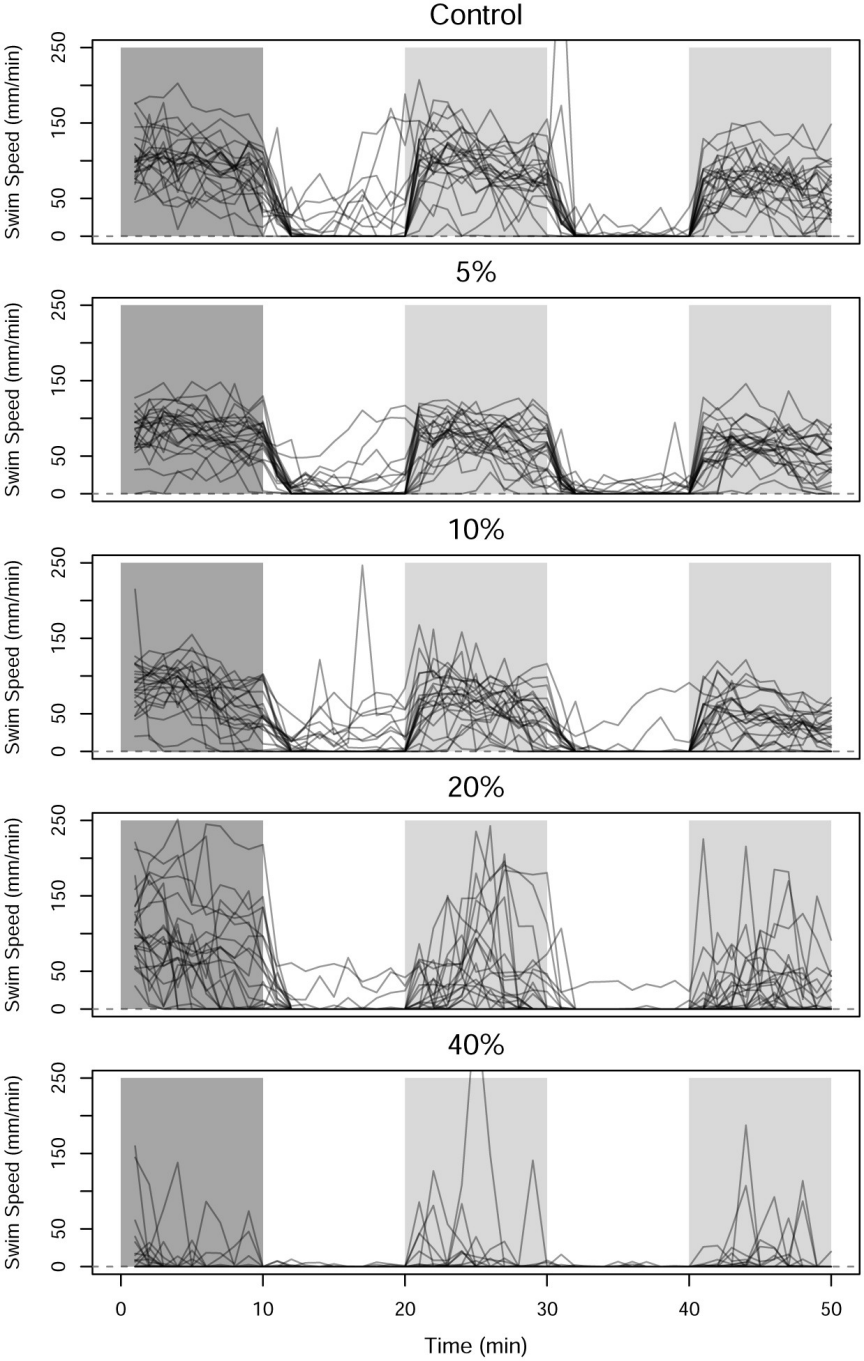
Pointwise observations of swim speed by concentration of 1-heptanol. The color of the background denotes the acclimation period (dark gray) and the alternating light (white) and dark (light gray) ten-minute periods.

The zebrafish are divided into five groups of twenty four and exposed to concentrations of 1-heptanol at either 0%, 5%, 10%, 20%, and 40% of their median lethal concentration (LC_50_). Fig 8 shows the observed swim speed of the 120 zebrafish, grouped by the concentration level. It is clear that the zebrafish are more active in the dark periods than the light periods. As the concentration of 1-heptanol increases, the zebrafish also appear to swim less overall.

**Fig 9:**
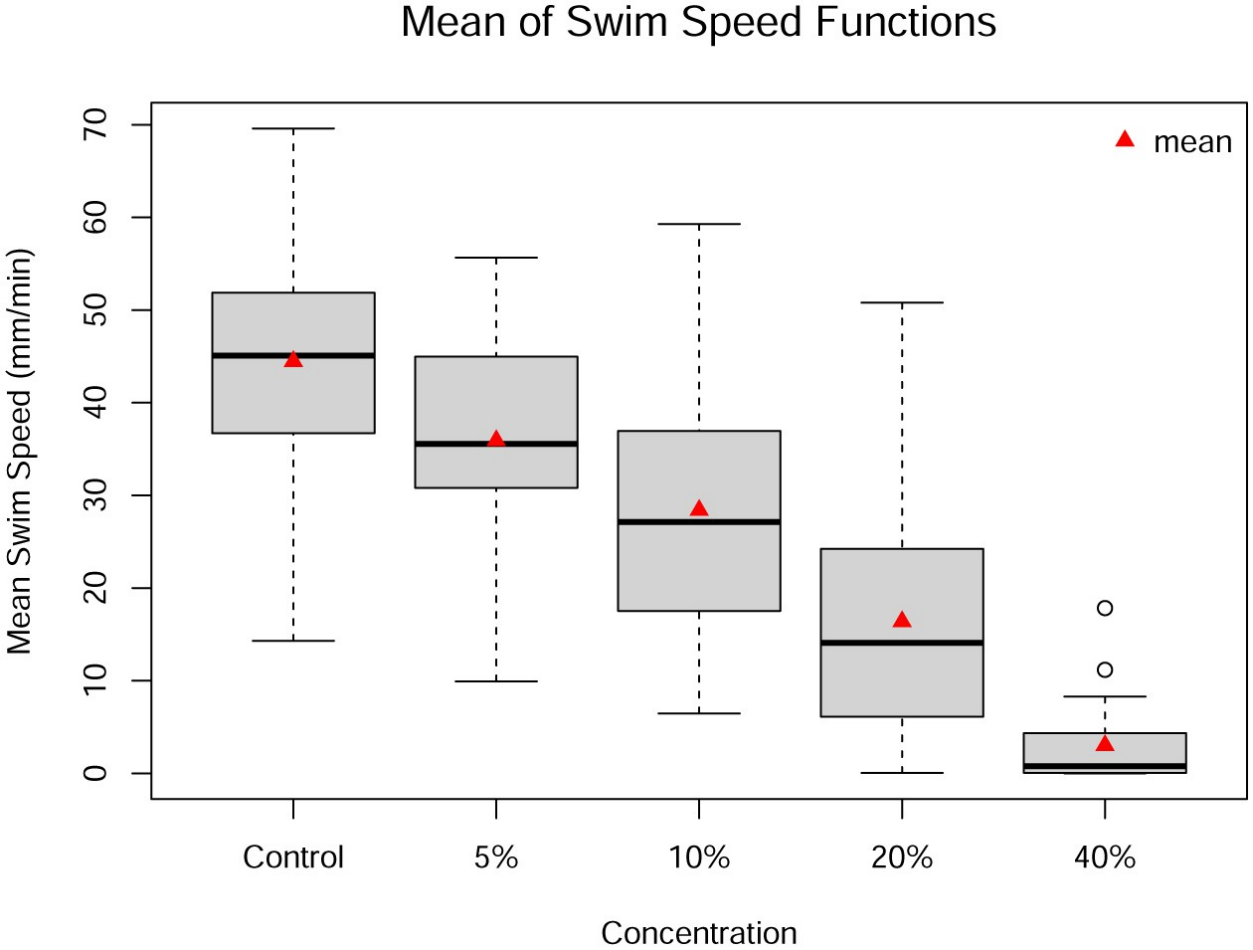
Boxplots of the averages of the functions for each concentration. Circles outside of the fences of the boxplots represent potential outliers.

For each individual fish, the mean swim speed across the light and dark periods is taken (excluding the acclimation period) and is plotted in Fig 9 by concentration. Each concentration level has a clearly different mean. This implies that a one-way univariate ANOVA is likely to be significant, but provides no information about where the differences occur in the domain.

### 4.1 Univariate and repeated measures ANOVA

The result of a univariate ANOVA applied to the means of each individual over the domain is shown in Table 6. As expected, the univariate ANOVA results in a low *p*-value, indicating a significant difference in the mean swim speed due to the concentration levels. Because the null hypothesis is rejected, the proper procedure is to perform a post hoc analysis to determine which pairs of concentration levels differ significantly, using the hypotheses in Eq 3. However, the post hoc analysis does not provide any additional information about where in the domain the differences occur.

**Table 6:** Univariate ANOVA results.

	Df	Sum Sq	Mean Sq	F-value	Pr(>F)
Concentration	4	25577.23	6394.31	48.51	<0.0001
Residual	115	15157.14	131.80		

Similarly, we apply repeated measures ANOVA to the data. Because all of the assumptions for repeated measures ANOVA are violated, any results obtained from the analysis are inconclusive, but Table 7 provides the results for illustration. All of the terms in the model, including the interaction, are significant. Because the assumptions are violated, pairwise comparisons for the interaction between concentration and time would be invalid. Furthermore, with forty repeated measurements, over 750 pairwise comparisons would be needed, and this is a prohibitive number to evaluate. Therefore, we do not include any post hoc analyses for the repeated measures ANOVA.

**Table 7:** Repeated measures ANOVA results.

	Df	Sum Sq	Mean Sq	F-value	Pr(>F)
Concentration	4	1023089.35	255772.34	48.51	<0.0001
Fish(Concentration)	115	606285.45	5272.05		
Time	39	2052231.27	52621.31	82.38	<0.0001
Concentration×Time	156	714188.17	4578.13	7.17	<0.0001
Residual	4485	2865001.94	638.80		

### 4.2 FANOVA application

**Fig 10:**
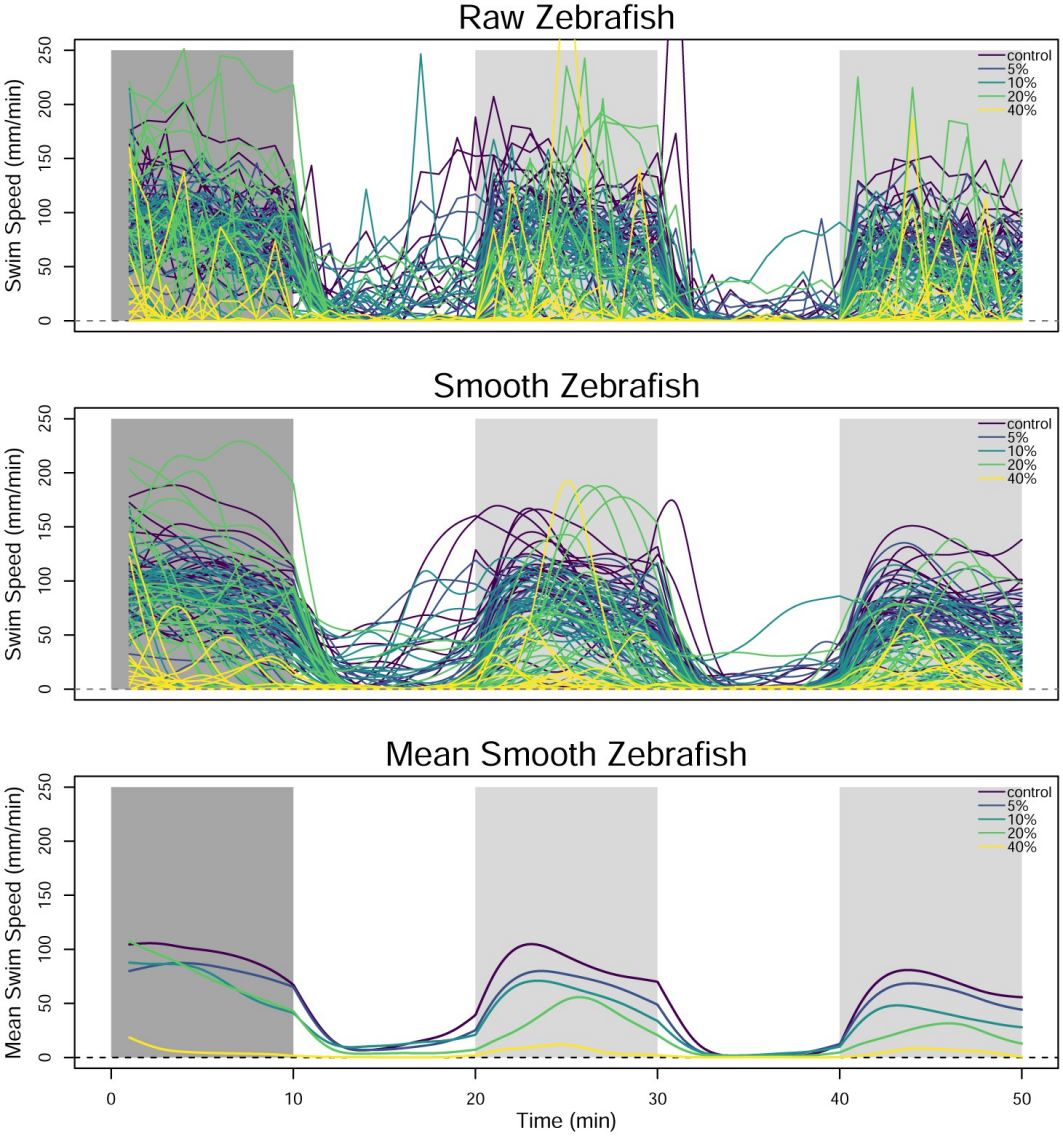
Raw observations of swim speed (top), smoothed observations of swim speed (center), and the functional averages of the smoothed swim speed curves for each concentration level (bottom).

Before applying FANOVA to the data, each function is smoothed using a cubic b-spline basis with sixty basis functions. The cubic b-spline basis set is selected because the data do not have a recognizable pattern. When the light turns on/off, the zebrafish display a rapid and immediate change in their swim pattern, so we build discontinuities into the smoothed functions at these times by allowing additional flexibility at these points. The basis functions are saturated, penalization is applied to the square of the second derivative, and the smoothing parameter is obtained through generalized cross validation to minimize the squared error between the smooth curves and discretized values. The smoothing results in the reduction of some large values that occur in the data, but the smooth curves also reduce the noise of the data. Another option would be to remove the functions with unusual values, and several methods for identification of functional outliers are available but are not explored here [38, 39]. Once the smoothing parameter is chosen, the smooth functions are then evaluated across a fine, evenly-spaced grid, and negative values are replaced with zeroes so that the swim speed functions remain non-negative across the domain. The results of the b-spline smoothing are shown in the center panel of Fig 10. To visualize the average swim speed for each concentration level, the average of the smooth swim speed curves for each concentration level is plotted in the bottom panel of Fig 10. In the two dark periods in particular, the average swim speed decreases as the concentration of 1-heptanol increases. In the light periods, there is only a small difference between the concentration levels.

**Fig 11:**
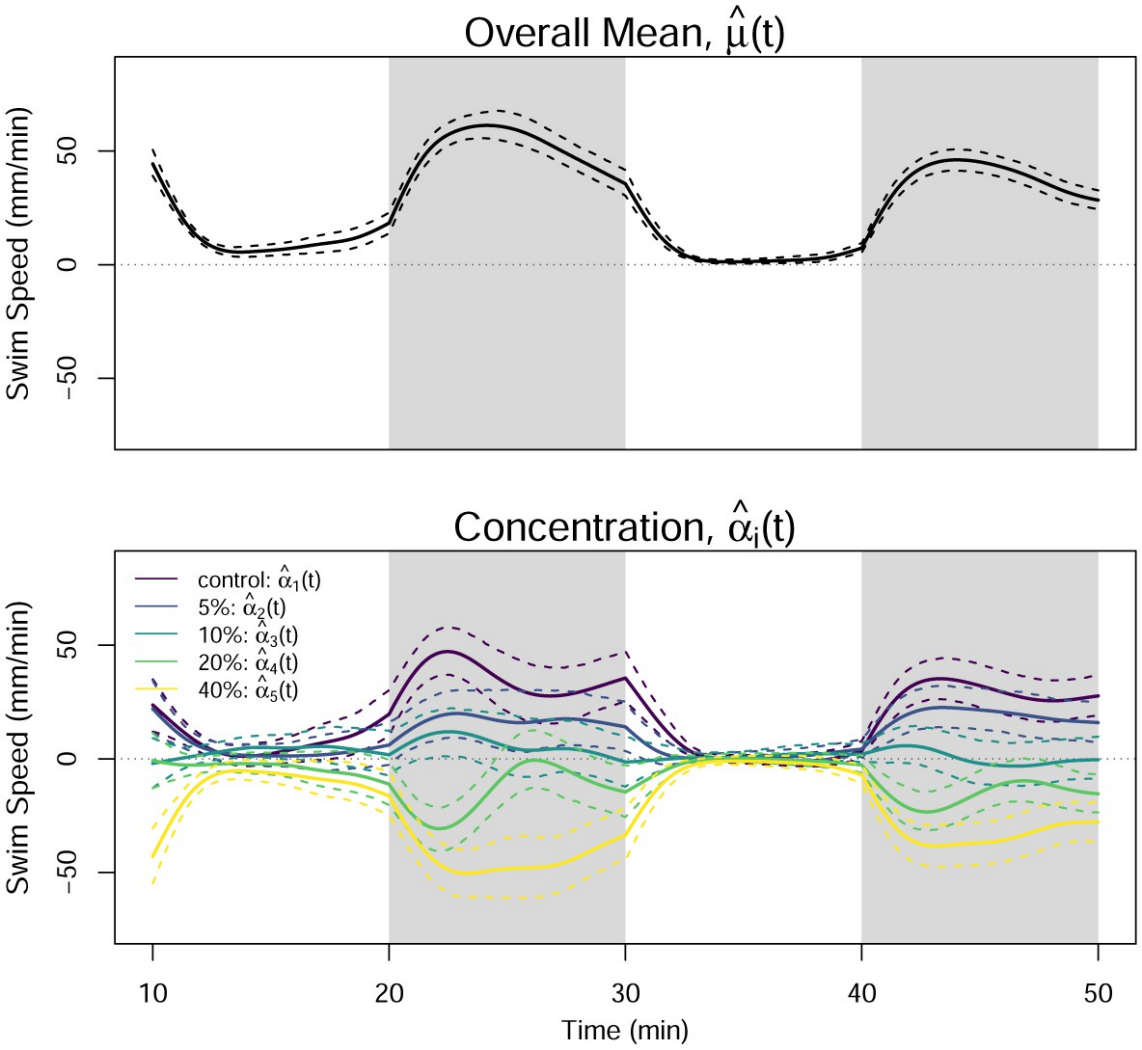
Estimated overall mean effect (top) and concentration effect (bottom). Dashed lines are 95% pointwise confidence intervals formed using 1000 bootstrap replicates.

One-way FANOVA is applied to the smoothed curves plotted in Fig 10, and the parameters in the model of Eq 22 are estimated. Fig 11 displays the estimated functional effects and 95% bootstrapped pointwise confidence intervals formed using the process described in Section 3.3. The overall mean effect illustrates the overall lightness aversion of the zebrafish, which is also clear in Figs 8 and 10. The concentration effects illustrate that higher concentrations result in lower swim speeds, particularly in the dark regions. There is less difference in the concentration effect during the light periods. Note that because we do not include the acclimation period in our analysis of the data, it is not included in this figure.

**Fig 12:**
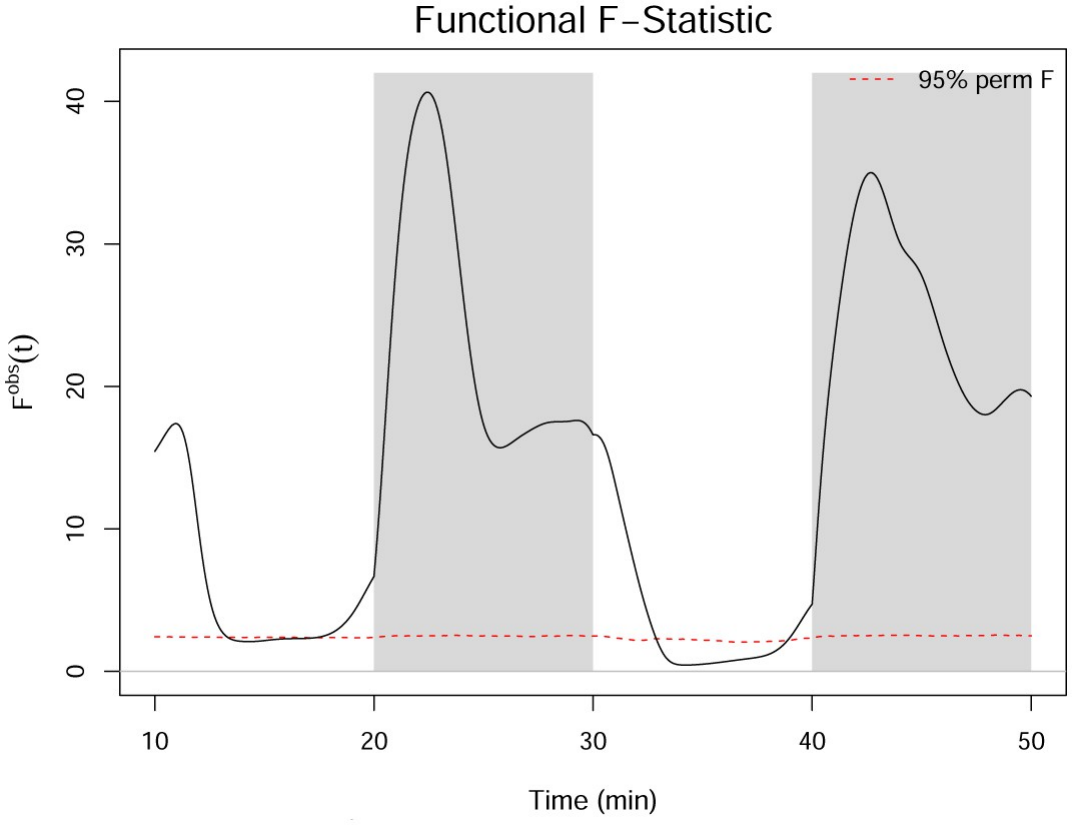
One-way FANOVA *F*^*obs*^(*t*) statistic applied to the smoothed dataset. The red dashed line indicates the 95% pointwise percentile calculated from the permutations.

**Fig 13:**
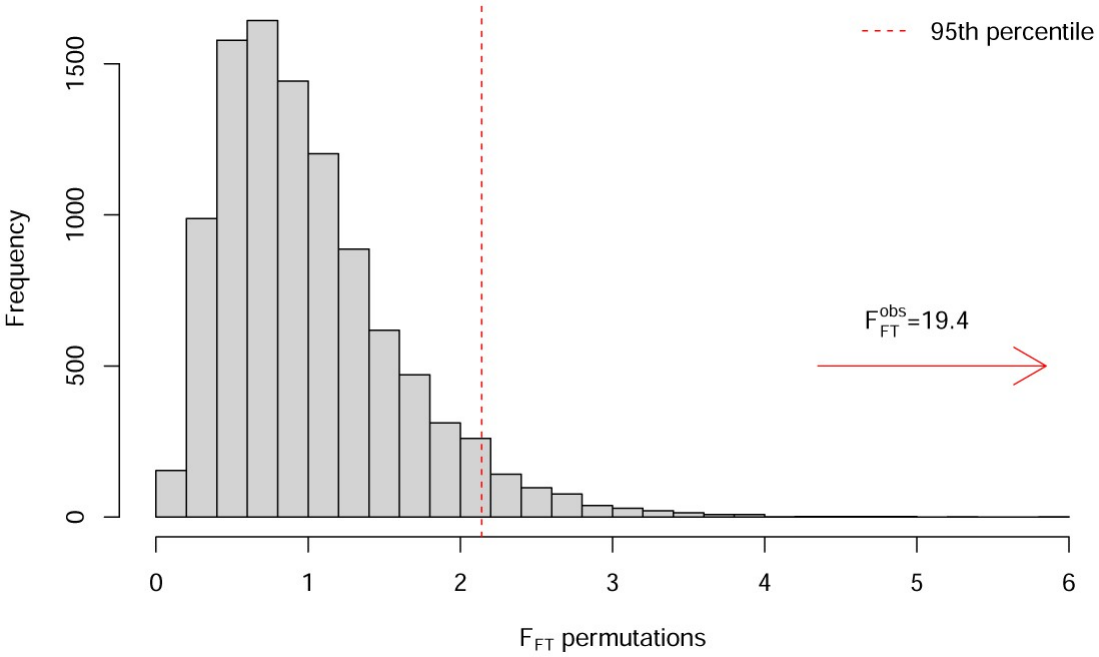
Histogram of 10,000 *F*_*FT*_ permutations. The red dashed line indicates the 95th percentile of the permutations.

The functional *F*-statistic for the hypotheses given in Eq 23 is computed according to Eq 27. Fig 12 shows the *F*^*obs*^(*t*) statistic from the one-way FANOVA results on the smoothed data. The *F*^*obs*^(*t*) statistic is particularly high in the dark regions, where the functions differ the most. We then perform the permutation test for the *F*_*FT*_ statistic described in Section 2.3.5 using 10,000 permutations. Fig 13 shows a histogram of the 10,000 permuted *F*_*FT*_ statistics, illustrating its sampling distribution under the null hypothesis. Using a significance level of *τ* = 0.05, the permuted critical value of *F*_*FT*_ is $F_{FT}^{crit}=2.14$, which is much smaller than the observed $F_{FT}^{obs}=19.39$. The *p*-value for the *F*_*FT*_ test is zero, indicating that 100% of the permuted statistics are below the critical value. In addition to concluding that significant differences in the mean functions exist across concentrations, FANOVA also identifies approximately where in the domain the differences occur.

#### 4.2.1 Post hoc analysis

**Fig 14:**
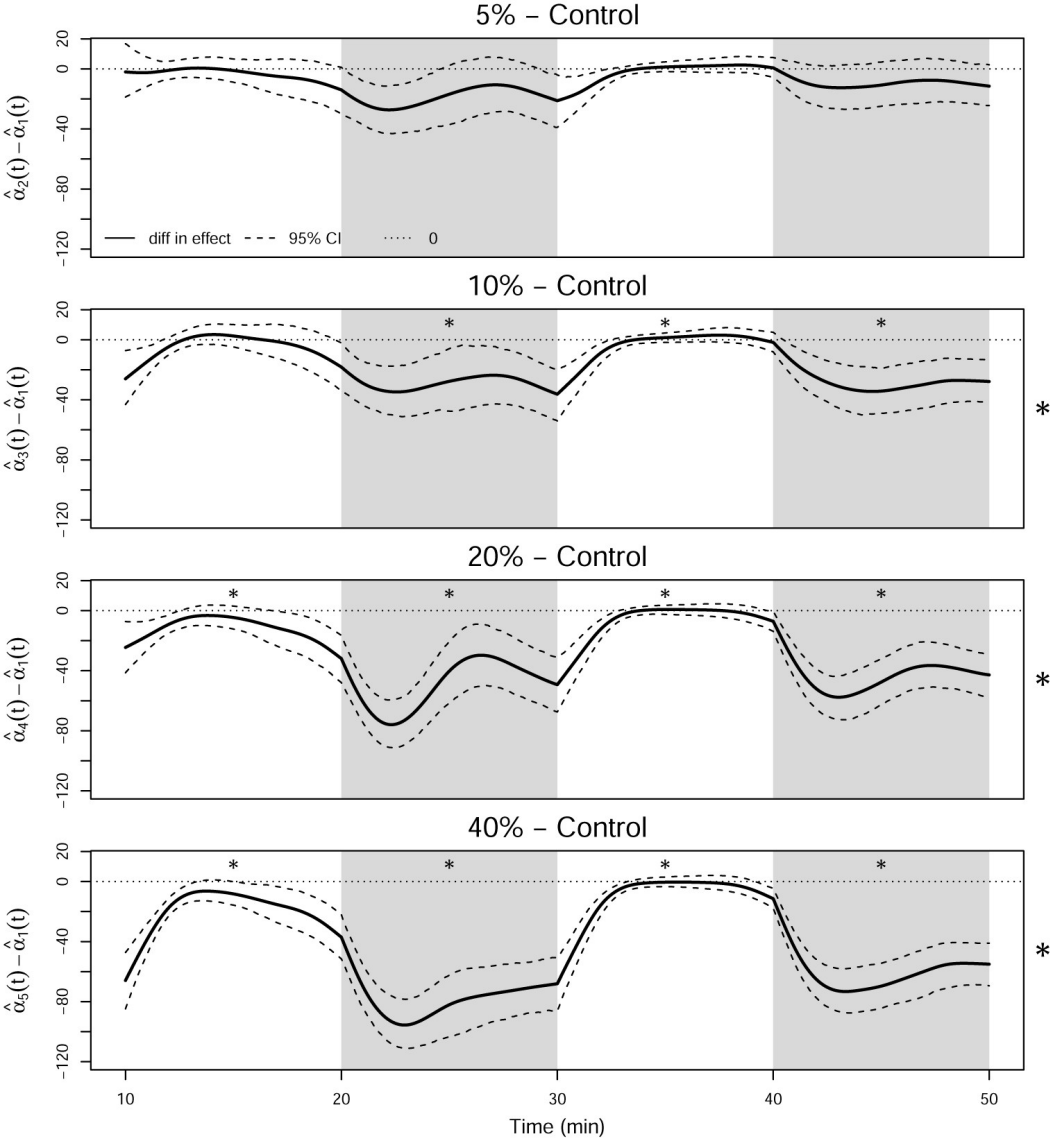
Functional post hoc contrasts for the concentration levels. The dashed line is the pointwise 95% confidence interval using 1000 bootstrap replicates. The asterisks on the right of the plots indicate significance of the *F*_*FT*_ test for the concentration effect across the entire domain, and the asterisks inside the plots indicate significance within the corresponding brightness period.

Because we reject *H*_0_, a post hoc analysis of the concentration levels to test the hypotheses given in Eq 24 are performed next with results in Table 8. We only compare each concentration level with the control group because the average effect of an increase in the concentration level on swim speed is negative, which can be seen in Fig 11. The tests employ a Bonferroni correction so that the significance level for each test is the nominal significance level (*τ* = 0.05) divided by the number of tests. The functional contrasts shown in Fig 14 are constructed by calculating ${\hat{\alpha }}_{i}\left(t\right)-{\hat{\alpha }}_{1}\left(t\right)$ with *i* ≠ 1 and forming 95% pointwise bootstrapped confidence intervals with 1000 replicates. We determine that there is a significant difference between the mean swim speeds of the control group and the groups with concentrations of 1-heptanol at 10%, 20%, and 40% of their LC_50_.

**Table 8:** The *F*_*FT*_ test results for the main effects and post hoc tests for the entire domain and each of the light/dark regions. For each region name, the letter refers to the brightness condition (light/dark), and the number corresponds to whether the region is the first or second light/dark period. In the “Decision” column, FTR means “fail to reject.”

Region	*H*_0_	$F_{FT}^{crit}$	$F_{FT}^{obs}$	*p*-value	Sig. Level	Decision
L1-D2: *t* ∈ [10, 50)	*α*_1_(*t*) = ⋯ = *α*_5_(*t*)	2.14	19.39	0.0000	*τ*	Reject
	*α*_1_(*t*) = *α*_2_(*t*)	0.36	0.26	0.0393	*τ*/4	FTR
	*α*_1_(*t*) = *α*_3_(*t*)	0.37	0.86	0.0001	*τ*/4	Reject
	*α*_1_(*t*) = *α*_4_(*t*)	0.37	2.35	0.0000	*τ*/4	Reject
	*α*_1_(*t*) = *α*_5_(*t*)	0.35	5.11	0.0000	*τ*/4	Reject
L1: *t* ∈ [10, 20)	*α*_1_(*t*) = *α*_3_(*t*)	0.46	0.22	0.0695	*τ*/12	FTR
	*α*_1_(*t*) = *α*_4_(*t*)	0.49	0.54	0.0028	*τ*/12	Reject
	*α*_1_(*t*) = *α*_5_(*t*)	0.50	1.60	0.0000	*τ*/12	Reject
D1: *t* ∈ [20, 30)	*α*_1_(*t*) = *α*_3_(*t*)	0.59	0.83	0.0003	*τ*/12	Reject
	*α*_1_(*t*) = *α*_4_(*t*)	0.59	2.60	0.0000	*τ*/12	Reject
	*α*_1_(*t*) = *α*_5_(*t*)	0.55	6.09	0.0000	*τ*/12	Reject
L2: *t* ∈ [30, 40)	*α*_1_(*t*) = *α*_3_(*t*)	0.50	0.87	0.0003	*τ*/12	Reject
	*α*_1_(*t*) = *α*_4_(*t*)	0.55	1.59	0.0000	*τ*/12	Reject
	*α*_1_(*t*) = *α*_5_(*t*)	0.54	2.89	0.0000	*τ*/12	Reject
D2: *t* ∈ [40, 50)	*α*_1_(*t*) = *α*_3_(*t*)	0.63	1.32	0.0000	*τ*/12	Reject
	*α*_1_(*t*) = *α*_4_(*t*)	0.63	3.22	0.0000	*τ*/12	Reject
	*α*_1_(*t*) = *α*_5_(*t*)	0.60	6.17	0.0000	*τ*/12	Reject

With the functional approach, it is also possible to explore subregions of the post-hoc contrasts to determine under which brightness conditions the differences occur. Note that because the 5% concentration level was not significantly different from the control group, no further comparisons are made. Thus, there are twelve comparisons, so the significance level for each test is *τ*/12. Table 8 shows that the 1-heptanol 20% (*α*_4_(*t*)) and 40% (*α*_5_(*t*)) LC_50_ groups are significantly different from the control group in all four subregions, while the 1-heptanol 10% (*α*_3_(*t*)) LC_50_ is significant in every region except for the first light region. While significance in the dark regions is expected, it is surprising to find significance in the light regions. This is likely due to the time it takes for the fish to slow down at the beginning of the light periods.

### 4.3 Startle response analysis

Some researchers are particularly interested in the change in swim speed in response to a change in conditions, called the startle response. There have been many approaches to analyzing the startle response, most of which involve performing univariate or repeated measures ANOVA for either the maximum swim speed post-stimulus [40, 41]; the average duration of the response [41]; or the average swim speed for short periods directly preceding and/or following a change in conditions [5, 18, 42, 43]. Sometimes, *t*-tests or nonparametric alternatives are performed for every time point against a baseline speed or control group [44, 45]. Another approach involves grouping swim behavior into speed thresholds that indicate bursting and/or freezing speeds [8, 15, 17]. Several researchers use univariate statistical methods to capture more information about the startle response, such as analyzing both the average swim speed and the net change in swim speed from the end of one period to the beginning of the following period [9, 46] or analyzing responsiveness, response latency, and maximum angular velocity during the startle [47]. All of this information is captured using the functional approach. Because the startle response occurs over some period of time and may last varying amounts of time for each individual, the functional representation of the observations provides a natural way to visualize and quantify it.

**Fig 15:**
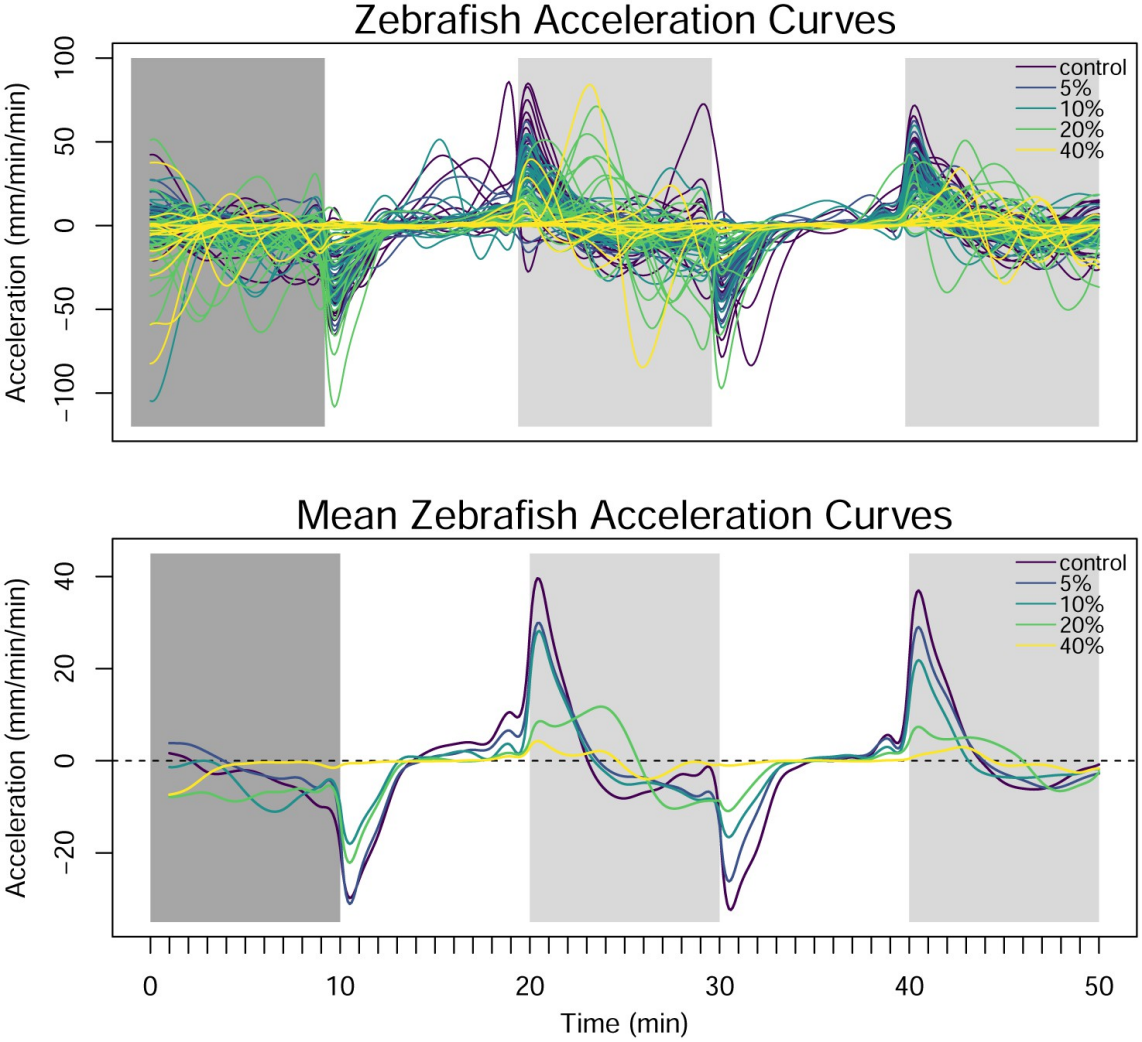
The first derivative of the smoothed swim speed curves (top), and the functional averages of the first derivative of the smoothed swim speed curves for each concentration level (bottom).

A novel way to analyze the startle response is to compute the acceleration function of the zebrafish. Given smoothed curves of swim speed, the acceleration can be found by taking their first derivative, as shown in the top panel of Fig 15. When the light is turned on, there is a downward spike in the acceleration, and conversely, there is an upward spike when time the light is turned off. The zebrafish appear to accelerate or decelerate for approximately three minutes after a change in the brightness conditions. To assess if the acceleration differs across the concentration levels, the average of the acceleration curves for each concentration level is plotted in the bottom panel of Fig 15. As the concentration of 1-heptanol increases, the strength of the average response of the zebrafish to the change in brightness decreases.

**Fig 16:**
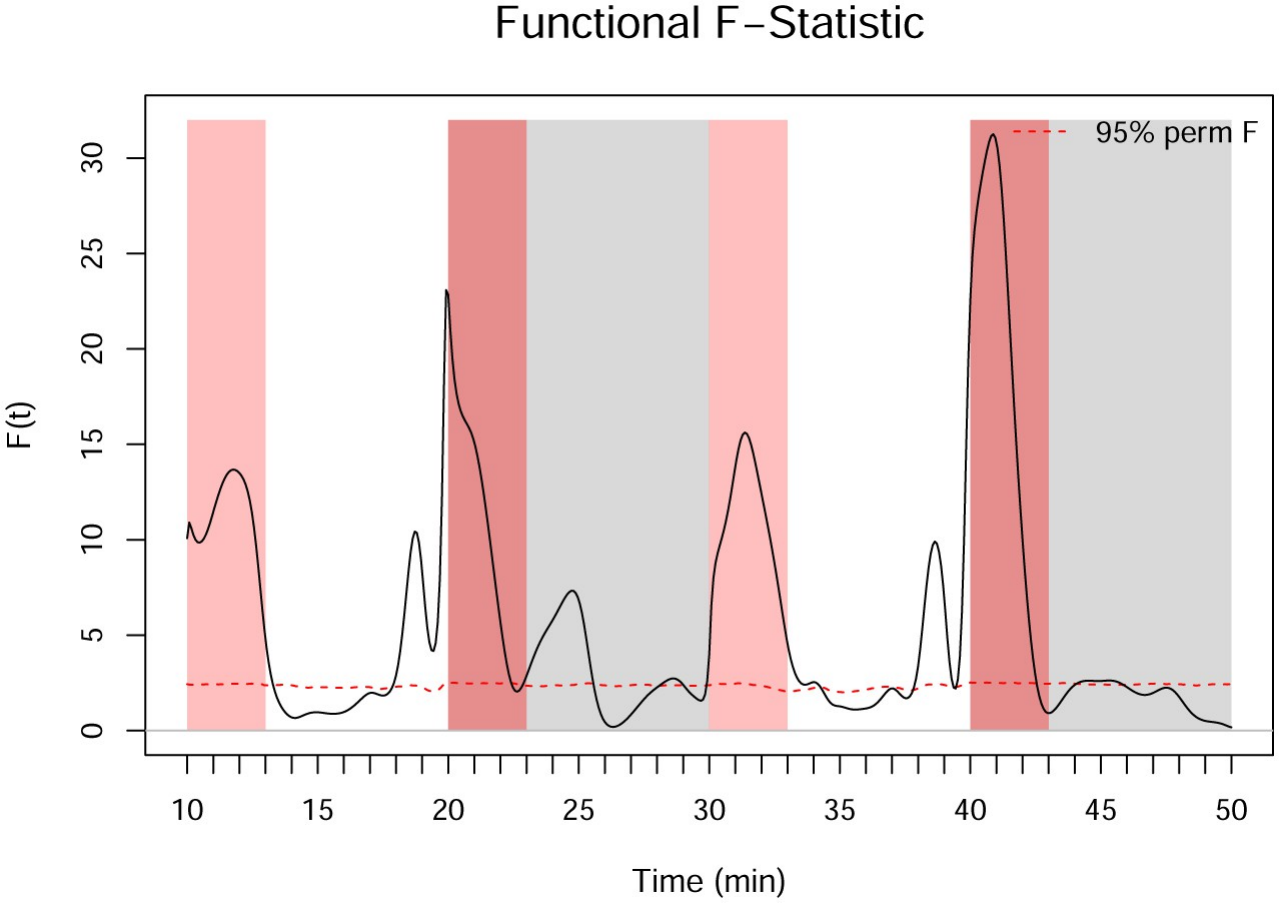
Functional *F*^*obs*^(*t*) statistic of the acceleration curves, where the red dashed line indicates the 95% pointwise percentile calculated from the permutations. Regions of interest for the startle response are represented in light pink shading for dark to light transitions and dark pink shading for light to dark transitions.

**Fig 17:**
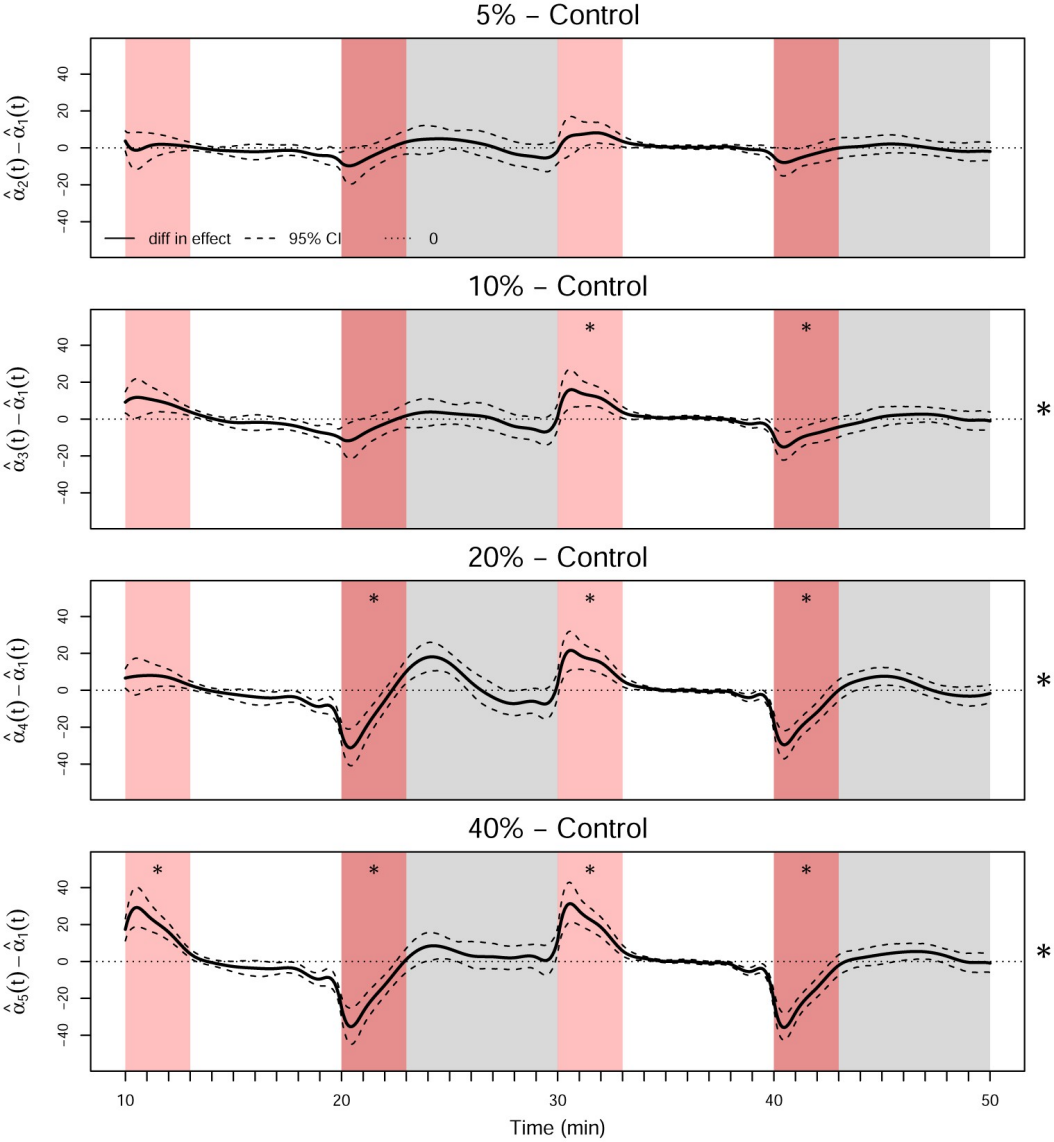
Functional post hoc contrasts for the acceleration curves for the concentration levels. The dashed line is the pointwise 98.75% confidence interval using 1000 bootstrap replicates. Regions of interest for the startle response are represented in light pink shading for dark to light transitions and dark pink shading for light to dark transitions. The asterisks indicate significance of the *F*_*FT*_ test for the concentration effect in the corresponding region.

One-way FANOVA can also be applied to the smooth acceleration curves to test for a significant difference in the acceleration of the groups for the three minutes after a change in brightness. The functional *F*-statistic for the entire domain is shown in Fig 16. Because the acclimation period is not relevant for this analysis, it is not included in the remaining figures in this section. There is a significant difference in the average acceleration across the concentration groups for approximately two to three minutes after the light turns on and a very strong spike in the *F*-statistic for two to three minutes after the light turns off. Thus, we perform post hoc analysis to determine which of the groups are significantly different from the control group during those periods. Complete results are given in Table 9. The contrasts shown in Fig 17 indicate that the group with the highest concentration of 1-heptanol has a significantly smaller startle response than the control group at the beginning of each period, which is shown in Table 9. The 20% LC_50_ concentration group has a significantly smaller startle response than the control group at the beginning of each period except for the first light period, and the 10% LC_50_ concentration group has a significantly smaller startle response than the control group at the beginning of the last two periods.

**Table 9:** The *F*_*FT*_ test results for the main effects and post hoc tests for the acceleration curves across the entire domain and the first three minutes of each of the light/dark regions. In the “Decision” column, FTR means “fail to reject.”

Region	*H*_0_	$F_{FT}^{crit}$	$F_{FT}^{obs}$	*p*-value	Sig. Level	Decision
L1-D2: *t* ∈ [10, 50)	*α*_1_(*t*) = ⋯ = *α*_5_(*t*)	1.50	7.94	0.0000	*τ*	Reject
	*α*_1_(*t*) = *α*_2_(*t*)	0.23	0.14	0.1086	*τ*/4	FTR
	*α*_1_(*t*) = *α*_3_(*t*)	0.23	0.39	0.0007	*τ*/4	Reject
	*α*_1_(*t*) = *α*_4_(*t*)	0.22	1.16	0.0000	*τ*/4	Reject
	*α*_1_(*t*) = *α*_5_(*t*)	0.23	1.76	0.0000	*τ*/4	Reject
L1: *t* ∈ [10, 13]	*α*_1_(*t*) = *α*_3_(*t*)	0.67	0.59	0.0068	*τ*/12	FTR
	*α*_1_(*t*) = *α*_4_(*t*)	0.66	0.31	0.0452	*τ*/12	FTR
	*α*_1_(*t*) = *α*_5_(*t*)	0.67	2.80	0.0000	*τ*/12	Reject
D1: *t* ∈ [20, 23]	*α*_1_(*t*) = *α*_3_(*t*)	0.60	0.28	0.0499	*τ*/12	FTR
	*α*_1_(*t*) = *α*_4_(*t*)	0.60	2.03	0.0000	*τ*/12	Reject
	*α*_1_(*t*) = *α*_5_(*t*)	0.62	2.88	0.0000	*τ*/12	Reject
L2: *t* ∈ [30, 33]	*α*_1_(*t*) = *α*_3_(*t*)	0.57	0.87	0.0008	*τ*/12	Reject
	*α*_1_(*t*) = *α*_4_(*t*)	0.58	1.58	0.0000	*τ*/12	Reject
	*α*_1_(*t*) = *α*_5_(*t*)	0.60	3.08	0.0000	*τ*/12	Reject
D2: *t* ∈ [40, 43]	*α*_1_(*t*) = *α*_3_(*t*)	0.58	0.95	0.0006	*τ*/12	Reject
	*α*_1_(*t*) = *α*_4_(*t*)	0.59	3.28	0.0000	*τ*/12	Reject
	*α*_1_(*t*) = *α*_5_(*t*)	0.57	4.74	0.0000	*τ*/12	Reject

The startle response is an important behavioral response of interest in PBR literature, and the functional approach provides researchers with a systematic approach to analyzing the startle response. By using FANOVA, we are able to analyze both the duration and magnitude of the startle response and determine differences in treatment groups over regions of the domain of varying lengths. This could offer a new approach for diagnostic evaluation of tipping points of chemical contaminants following exposure.

## 5 Conclusion

Zebrafish PBR studies are of great value to toxicological and pharmacological research. However, the typical statistical methods applied in PBR studies prevents researchers from fully exploiting all of the information contained in the data. The most common approach, univariate ANOVA for each individual’s average across the entire domain, reduces each individual’s values to a single value and can produce untrustworthy results due to violation of the independence of observations within each individual. Even repeated measures ANOVA, the method used in PBR literature that best accounts for the temporal dependence in observations, has limitations in its interpretability, practical use, and assumptions that may not be met. To overcome these problems, we suggest the use of functional ANOVA, a method that naturally retains the functional structure of PBR data and produces valid, fast, and interpretable results. The FANOVA presented in this paper only requires that the observations are independent and can be used to analyze the response over the entire domain, detecting which regions of the domain have the largest differences. Because of the smooth functional representation of the data, it becomes possible to take the derivative of the speed functions to find the acceleration curves, so researchers can analyze the startle response in addition to the original measurement. Furthermore, FANOVA can be applied to any subregion of the domain, so researchers can apply FANOVA to the regions where the startle responses occur.

In this paper, we present a framework for implementing one-way FANOVA that has minimal assumptions and demonstrate how to apply it. We compare the results to those obtained from univariate and repeated measures ANOVA, illustrating the ways FANOVA overcomes their limitations. In order to facilitate the use of FANOVA in future PBR research, the R code and data used to perform the analyses in this paper and to create every figure and table are provided on the Harvard Dataverse data repository [37].

It is possible to extend this work beyond light/dark transition tests to other responses that are considered functional. Many studies that examine zebrafish also collect data on spontaneous movement/tail flexes [21, 48–50], bioaccumulation [51], and/or hatching rate [11]. Even when the light/dark transition test is not used, general locomotor data are commonly collected to assess the behavioral response of zebrafish [3, 15, 52]. While we have not performed an exhaustive comparison of all statistical methods historically used on these types of data, we argue that functional methods can and should be used. Any data that are collected repeatedly for the same subjects over a continuous domain can benefit from a functional approach.
